# Identification of murine gammaherpesvirus 68 miRNA-mRNA hybrids reveals miRNA target conservation among gammaherpesviruses including host translation and protein modification machinery

**DOI:** 10.1371/journal.ppat.1007843

**Published:** 2019-08-08

**Authors:** Whitney L. Bullard, Mehmet Kara, Lauren A. Gay, Sunantha Sethuraman, Yiping Wang, Shreya Nirmalan, Alim Esemenli, April Feswick, Brett A. Hoffman, Rolf Renne, Scott A. Tibbetts

**Affiliations:** Dept. of Molecular Genetics and Microbiology, UF Health Cancer Center, University of Florida, Gainesville, Florida, United States of America; University of Southern California, UNITED STATES

## Abstract

Gammaherpesviruses, including the human pathogens Epstein-Barr virus (EBV) and Kaposi’s sarcoma-associated herpesvirus (KSHV), establish lifelong latent infection in B cells and are associated with a variety of tumors. In addition to protein coding genes, these viruses encode numerous microRNAs (miRNAs) within their genomes. While putative host targets of EBV and KSHV miRNAs have been previously identified, the specific functions of these miRNAs during *in vivo* infection are largely unknown. Murine gammaherpesvirus 68 (MHV68) is a natural pathogen of rodents that is genetically related to both EBV and KSHV, and thus serves as an excellent model for the study of EBV and KSHV genetic elements such as miRNAs in the context of infection and disease. However, the specific targets of MHV68 miRNAs remain completely unknown. Using a technique known as qCLASH (quick crosslinking, ligation, and sequencing of hybrids), we have now identified thousands of Ago-associated, direct miRNA-mRNA interactions during lytic infection, latent infection and reactivation from latency. Validating this approach, detailed molecular analyses of specific interactions demonstrated repression of numerous host mRNA targets of MHV68 miRNAs, including *Arid1a*, *Ctsl*, *Ifitm3* and *Phc3*. Notably, of the 1,505 MHV68 miRNA-host mRNA targets identified in B cells, 86% were shared with either EBV or KSHV, and 64% were shared among all three viruses, demonstrating significant conservation of gammaherpesvirus miRNA targeting. Pathway analysis of MHV68 miRNA targets further revealed enrichment of cellular pathways involved in protein synthesis and protein modification, including eIF2 Signaling, mTOR signaling and protein ubiquitination, pathways also enriched for targets of EBV and KSHV miRNAs. These findings provide substantial new information about specific targets of MHV68 miRNAs and shed important light on likely conserved functions of gammaherpesvirus miRNAs.

## Author summary

Gammaherpesviruses, including the human pathogens Epstein-Barr virus (EBV) and Kaposi’s sarcoma-associated herpesvirus (KSHV), establish lifelong infections and are associated with a variety of tumors. These viruses encode numerous molecules called microRNAs (miRNAs) within their genomes, which target and suppress the products of specific genes within infected host cells. However, the function of these miRNAs during *in vivo* infection is largely unknown. Murine gammaherpesvirus 68 (MHV68) is a natural pathogen of rodents that is genetically related to both EBV and KSHV, and thus serves as an excellent model for the study of EBV and KSHV. Here, we describe the identification and validation of thousands of new MHV68 miRNA targets. Notably, 86% of the MHV68 miRNA targets identified were shared with either EBV or KSHV, and 64% were shared among all three viruses. Further analyses revealed enrichment of cellular pathways involved in protein synthesis and protein modification, including pathways also enriched for targets of EBV and KSHV miRNAs. These findings provide substantial new information about specific targets of MHV68 miRNAs and shed important light on likely conserved functions of gammaherpesvirus miRNAs.

## Introduction

Gammaherpesviruses are a family of large double-stranded DNA viruses that establish lifelong latent infections in their hosts. This group includes the human pathogens Epstein-Barr virus (EBV) and Kaposi’s sarcoma-associated herpesvirus (KSHV), as well as murine gammaherpesvirus 68 (MHV68, MuHV-4, γHV68). These viruses gain an initial foothold in the host by replicating in epithelial cells at the site of inoculation, and then subsequently establish lifelong latent infection in the periphery in circulating B cells. Although infection with gammaherpesviruses is typically asymptomatic, virus-driven malignancies such as B cell lymphomas may manifest during chronic infection, particularly within the setting of immunocompromise.

In addition to encoding an array of proteins with conserved functions, EBV, KSHV, and MHV68 all encode multiple microRNAs (miRNAs) [1–5]. miRNAs are 21–23 nt noncoding RNAs that are critical regulators of gene expression [6]. miRNAs integrate into the RNA-induced silencing complex (RISC), and are directed to target mRNAs through partial sequence complementarity [6]. In the context of RISC, the miRNA-mRNA binding interaction can result in mRNA silencing through translational repression or cleavage of the mRNA target [7,8].

Viral miRNAs are thought to play roles in diverse biological processes to promote viral persistence within the host. EBV and KSHV miRNAs are expressed in latently infected primary human cells and have been proposed to be important for the establishment and maintenance of chronic infection. EBV and KSHV encode 44 and 25 mature miRNAs, respectively [1–3,5]. Many host targets for EBV and KSHV miRNAs have been identified and validated, including host transcripts that code for proteins involved in cell cycle progression, apoptosis, and immune regulation [9–11]. For example, multiple EBV and KSHV miRNAs have been shown to target and decrease the expression of caspase-3, a key effector of apoptosis [12–14]. Likewise, both EBV and KSHV miRNAs have been shown to target MICB, a stress-induced ligand for the activating natural killer (NK) cell receptor NKG2D [15]. Numerous other putative targets of EBV and KSHV miRNAs have been identified in tumor cells [16–21]; however, the true *in vivo* function of most EBV and KSHV miRNAs are largely unknown due to the complex nature of studying virus-host interactions *in vivo* and the strict species specificity of these viruses.

Murine gammaherpesvirus 68 is a natural pathogen of rodents [22] that is genetically and pathogenically related to both EBV and KSHV [23–25]. Like the human gammaherpesviruses, MHV68 establishes lifelong latent infection in B cells [26–28], and results in the development of lymphoproliferative disease and B cell lymphoma [29,30]. MHV68 encodes up to 28 mature miRNAs, many of which are abundantly expressed during long-term latency and in lymphoproliferative lesions and tumors [2,31–34]. While the specific targets of the MHV68 miRNAs have not yet been determined, we have previously demonstrated the biological importance of these miRNAs during *in vivo* infection: a MHV68 mutant lacking expression of all 28 miRNAs is significantly attenuated for latency establishment and displays complete absence of pathology in a lethal pneumonia model [34]. Interestingly, similar combinatorial miRNA mutant viruses display increased number of infected cells in immunodeficient mice during early infection [35] and in wild-type mice during long-term infection [36], suggesting that the MHV68 miRNAs may fine-tune the delicate balance between latency and reactivation throughout chronic infection and viral pathogenesis.

In work described here, we utilized a powerful new technique known as qCLASH (quick crosslinking, ligation, and sequencing of hybrids) to reveal the precise mRNA targets of MHV68 miRNAs [37–39]. Unlike crosslinking and immunoprecipitation (CLIP) approaches, which require bioinformatic identification of putative mRNA targets within a coupled data set following sequencing of separate miRNA and mRNA libraries, qCLASH directly identifies specific miRNA-target interactions through the sequencing of ligated miRNA-target mRNA hybrids. Like CLIP, the basis of CLASH is the purification of crosslinked RISC-RNA complexes by immunoprecipitation of Argonaute-2 (Ago-2), a major protein component of RISC. However, in contrast to CLIP protocols, CLASH utilizes RNA ligase to ligate miRNAs to their Ago-protected binding partners. Libraries generated from the resultant miRNA-mRNA hybrids are then subjected to high throughput sequencing. Sequencing results are stringently processed using the bioinformatic pipeline Hyb [40], which identifies miRNA and mRNA sequences and complementarity sequences within each hybrid. Recently, qCLASH, a modified version of this procedure which allows for a reduced amount of input material, was used to define precise targets of KSHV miRNAs [37,41].

Here, we utilized the qCLASH approach to identify host mRNA targets of MHV68 miRNAs during lytic infection, latent infection, and reactivation from latency. Cumulatively, we defined 2,493 unique, high-confidence MHV68 miRNA-host mRNA interactions. Follow-up molecular studies validated specific repression of individual targets. Analysis of host pathways targeted by MHV68 miRNAs revealed a high number of targets shared with EBV and/or KSHV miRNAs, including numerous shared targets within host translation and protein modification pathways.

## Materials and methods

### Ethics statement

This study was conducted in accordance with all institutional and federal guidelines. All animal protocols were approved by the Institutional Animal Care and Use Committee at the University of Florida (protocols 201609615 and 201708626).

### Cell culture and virus infections

NIH 3T12 murine fibroblasts (ATCC, CCL-164) were maintained in Dulbecco’s modified Eagle’s medium, DMEM (Corning, 11013CM) supplemented with 10% heat inactivated fetal bovine serum (FBS) and 1X penicillin/streptomycin (pen/strep; Corning, 30002CI). HE2.1 B cells (generated by Dr. Craig Forrest, provided by Dr. Laurie Krug) were maintained in RPMI 1640 medium (Corning, MT10040CM) supplemented with 10% FBS, 1X pen/strep (Corning, 30002CI), and 50μM 2-mercaptoethanol. HE2.1 cells were cultured in the presence of 300 μg/mL hygromycin as described previously [42]. For preparation of lytic samples, NIH 3T12 cells were infected with WT MHV68 at MOI of 5 and incubated at 37°C. Lytically infected cells were harvested 16 hours post infection (hpi). For preparation of latent samples, HE2.1 cells, a B lymphocyte cell line which is latently infected with WT MHV68, were collected in log phase growth. For preparation of reactivation samples, HE2.1 cells were treated with tetradecanoylphorbol acetate (TPA) at a concentration of 20 ng/mL and incubated at 37°C. Reactivated cells were harvested 16 hours after TPA treatment.

### qCLASH

qCLASH was performed on lytically infected NIH 3T12 cells, latently infected HE2.1 B cells, and TPA treated HE2.1 B cells, each in triplicate. The qCLASH analysis was performed as described previously by Gay et al. [37], but with modifications. Briefly, for each condition, 5.0x10^7^ cells were collected, washed twice in 1X PBS, resuspended in 10 mL 1X PBS, and transferred to a cell culture dish on ice. Cells were UV-irradiated, then cell pellets were frozen at -80°C. Protein G beads Dynabeads (Invitrogen, 10004D) were washed and then resuspended in AffiniPure Rabbit Anti-Mouse IgG (Jackson ImmunoResearch, 315-005-008). After washing, beads were resuspended in 2A8 anti-Ago antibody (generously provided by Dr. Zissimos Mourelatos). Cell lysates were resuspended in Lysis Buffer and incubated with RQ1 DNase (Promega, M610A). Lysate was centrifuged, and supernatant was incubated with RNAse T1. Prepared lysate was incubated with antibody-coated beads, then beads resuspended in Lysis Buffer containing RNase T1. Following high stringency washes, phosphorylation and intermolecular ligation was performed using T4 PNK and T4 RNA Ligase. Subsequently, dephosphorylation and 3’ linker addition were performed using Alkaline Phosphatase (Roche, 10713023001), then T4 RNA Ligase 2 truncated K227Q (NEB, M0351S) with miRCat-33 3’ linker (5’-TGGAATTCTCGGGTGCCAAGG-3’). Ago/RNA complexes were then eluted, proteins degraded by proteinase K (Roche, 03115887001) treatment, and RNA was extracted using Phenol/Chloroform/Isoamyl Alcohol (25:24:1).

For library preparation, RNA was incubated with T4 PNK mixture, then T4 RNA Ligase with 5’ RNA linker. RNA was extracted with Phenol/Chloroform/Isoamyl Alcohol, then RNA was resuspended in RT buffer containing reverse transcription primer (Illumina TruSeq Small RNA Sample Prep Kits RTP). Reverse transcription was carried out using SuperScript III (Invitrogen, 18080093). PCR was performed using 2x Phusion High-Fidelity Master Mix plus Primer 1 (Illumina TruSeq Small RNA Sample Prep Kits RP1), and Index Primers 1, 2, or 3 (Illumina TruSeq Small RNA Sample Prep Kits RPI1, RPI2, or RPI3). The resulting DNA libraries were separated on a 2% agarose gel, and regions corresponding to 175–300 bp were excised. DNA was gel purified from gel slices using the NucleoSpin Gel and PCR Clean-Up Kit (Clontech, 740609.250) according to the manufacturer’s instructions.

### Sequencing and bioinformatic analysis

qCLASH libraries were sequenced on a HiSeq 2500 with a read length of 100 bases. The raw sequences were pre-processed with Trimmomatic [43] to remove adapter sequences and then analyzed with Hyb, a bioinformatics pipeline developed specifically for the analysis of CLASH data [40]. Determination of base-pairing along the length of the miRNA, categorization of miRNA seed-pairing and 3’ end pairing, and determination of mRNA transcript region origin were all carried out with custom scripts adapted from qCLASH scripts for KSHV miRNAs (available at the GitHub page: http://github.com/RenneLab/qCLASH-Analysis).

### Luciferase assays

Regions of 500–1000 bp flanking the miRNA binding site of select genes were PCR amplified from NIH 3T12 cDNA using Q5 High-Fidelity DNA Polymerase (NEB, M0491S) according to the manufacturer’s instructions. The resulting PCR fragments were cloned into pmirGLO Dual-Luciferase miRNA Target Expression Vector (Promega, E1330) using Gibson Assembly Cloning Kit (NEB,E5510S) according to the manufacturer’s instructions. Minimal miRNA binding sites (approximately 30 bp) were generated by annealing complementary oligos in annealing buffer (10mM Tris, pH 7.5, 50mM NaCl, and 1mM EDTA) at 100°C for 10 min, then incubating overnight at room temperature. The annealed oligos were cloned into the SacI and XbaI sites in the pmirGLO Vector using T4 DNA Ligase (NEB, M0202S) according to the manufacturer’s instructions. A list of all primers used are listed in **S1 Table**. For luciferase assays, 1.0x10^4^ NIH 3T12 cells were plated per well of a 96-well plate and incubated overnight. Culture medium was removed and 50 μL transfection mixture was added to each well containing: 0.3 μL Lipofectamine-2000, 1 μL 50ng pmirGLO Plasmid, 0.5 μL 5 μM mirVana custom miRNA mimic (Life Technologies), 48.2 μL Opti-MEM Reduced Serum Medium (ThermoFisher, 11058021). Cells were then incubated with transfection mixture for 4 hours. 150 μL complete DMEM was added to each well and incubated overnight. Luciferase assays were performed with the Dual-Glo Luciferase Assay System (Promega, E2940) according to the manufacturer’s instructions. All luciferase assays were performed in biological triplicates and technical quadruplicates.

### Quantitative reverse transcription PCR (qRT-PCR)

Total RNA was obtained using Qiagen RNeasy Mini Kit (Qiagen, 74104) according to the manufacturer’s instructions. First-strand cDNA was synthesized from 1μg of total RNA using the NEB ProtoScript II Reverse Transcriptase (NEB, M0368S) per the manufacturer’s instructions. Quantification of selected genes were performed on an iCycler with an iQ5 multicolor real-time PCR detection system (Bio-Rad Laboratories, Hercules, CA). The reaction mixture contained 5 pmol forward and reverse primer, 2x iQ SYBR green super mix (Bio-Rad Laboratories), and 2 μl of template cDNA. Standard curves were prepared for each gene using 10-fold dilutions of a known quantity (300 ng/μL) of cDNA from 3T12 Cells. The quantities were calculated using iQ5 optical detection system software. Each sample was normalized to GAPDH mRNA. The primer sequences utilized in this analysis are listed in **S2 Table**. All qRT-PCR assays were performed in biological and technical triplicates.

### Western blotting

Approximately 1x10^6^ NIH 3T12 cells per sample were resuspended in 150 μL Lysis Buffer (150 mM NaCl, 1% NP-40, 50 mM Tris, pH 8.0, and 1X complete mini EDTA-free protease inhibitor) and stored at -80°C. A 1:1 mixture lysate and 2X Loading Buffer (100 mM Tris-HCl, pH 6.8, 4% SDS, 20% Glycerol, 0.05% Bromophenol Blue, and 10% 2-mercaptoethanol). Approximately 10 μg of total protein was then separated by sodium dodecyl sulfate polyacrylamide gel electrophoresis (SDS PAGE 10% gel), transferred to a nitrocellulose membrane and probed with antibodies directed to β-actin (Cell Signaling, 8H10D10), ARID1A (Novus Biologicals, NB100-55334), CTSL (R&D Systems, AF1515), EWSR1 (Abcam, EPR4647), IFITM3 antibody (R&D Systems, AF3377), or FOXJ3 antibody (R&D Systems, AF5786). Bound antibodies were detected by HRP-conjugated secondary goat anti-rabbit (Southern Biotech, 4050–05), goat anti-mouse (Southern Biotech, 1010–05), or rabbit anti-goat (Southern Biotech, 6160–05), then visualized by enhanced chemiluminescence. Western blots were performed in triplicates.

### ^35^S-methionine labeling assays

NIH 3T12 cells were infected at MOI 5 or MOI 10 and ^35^S labeling was carried out at either 5 or 10 hours post infection. For ^35^S labeling, infected cells were washed 3X with methionine- and cysteine-free Dulbecco’s modified Eagle’s medium (Thermo Fisher, 21013024) and were then incubated in met-cys-free DMEM for 1 hour at 37°C. Media was removed from cells and incubated with Met-Cys-Free DMEM containing 0.1 mCi/mL ^35^S-methionine (Perkin Elmer, NEG772007MC) for 30 minutes at 37°C. The labeling was stopped by addition of DMEM containing 10% FBS. Media was then removed, cells washed 3X with DMEM containing 10% FBS, and then washed 3X with 1X PBS. Cells were resuspended in lysis buffer and stored at -80°C. Equal amounts of total protein was then separated by sodium dodecyl sulfate polyacrylamide gel electrophoresis (SDS-PAGE 10% Gel) and the gel was dried using a gel drying kit (Promega, V7120) according to the manufacturer’s instructions. Dried gel was then exposed to film for 24 hours and developed using a Kodak X-OMAT 2000 film developer.

### Host pathways targeted by MHV68 miRNAs

Pathway data sets were analyzed in the context of canonical pathways generated by Ingenuity Pathway Analysis (IPA) (Qiagen; https://www.qiagenbio-informatics.com/products/ingenuity-pathway-analysis). To determine if any major cellular pathways are specifically targeted by MHV68 miRNAs, we analyzed the 2,493 genes present in two of three qCLASH biological replicates using IPA core analysis. The most significantly enriched canonical pathways were defined by IPA, and individual pathways were selected for visual representation here, with minor editing to clarify labels.

### Mice infections and flow cytometry

For flow cytometry-based sorting of infected cells, mice were infected with 10^4^ PFU MHV68-H2bYFP, a phenotypically wild-type virus that expresses eYFP under control of the H2b promoter [44]. At 16 dpi, splenocytes were prepared and blocked as described above. Cells were then stained with APC rat anti-mouse CD4 at 1:200 (BD Biosciences, 553051), APC rat anti-mouse CD8α at 1:200 (BD Biosciences, 553035), APC rat anti-mouse CD14 at 1:100 (BD Biosciences, 560634), and APC-Cy7 rat anti-mouse CD19 at 1:200 (BD Biosciences, 557655). Infected B cells (CD4^**-**^CD8^**-**^CD14^**-**^CD19^**+**^YFP^**+**^) and non-infected B cells (CD4^**-**^CD8^**-**^CD14^**-**^CD19^**+**^YFP^**-**^) were sorted using a BD FACSAria II flow cytometer (BD Biosciences). Sorted cells were immediately subjected to RNA extraction using an RNAqueous-Micro kit (Ambion, AM1931) prior to qRT-PCR analyses.

## Results

### qCLASH identifies MHV68 miRNA-host mRNA hybrids during latency, reactivation and lytic infection

To define the Ago-associated binding interactions between MHV68 miRNAs and cellular mRNAs, we performed a modified version of CLASH called quick CLASH (qCLASH) [37]. To assess miRNA targeting during latent infection, reactivation from latency, and lytic infection, three biological replicates were prepared from the MHV68+ B cell line HE2.1 [42], TPA-treated HE2.1 cells, and MHV68-infected NIH 3T12 fibroblast cells. At time of harvest, cells were crosslinked and then Ago complexes were precipitated. Following RNA-RNA ligation, RNA hybrids were eluted and cDNA libraries were prepared and sequenced. Each library yielded between 10 and 20 million reads, which were bioinformatically analyzed using the Hyb program [40] to identify any RNA-RNA chimeras that included any combination of host miRNA, viral miRNA, host mRNA, viral mRNA, or host lncRNA (sequences containing ribosomal RNAs were filtered).

**Fig 1:**
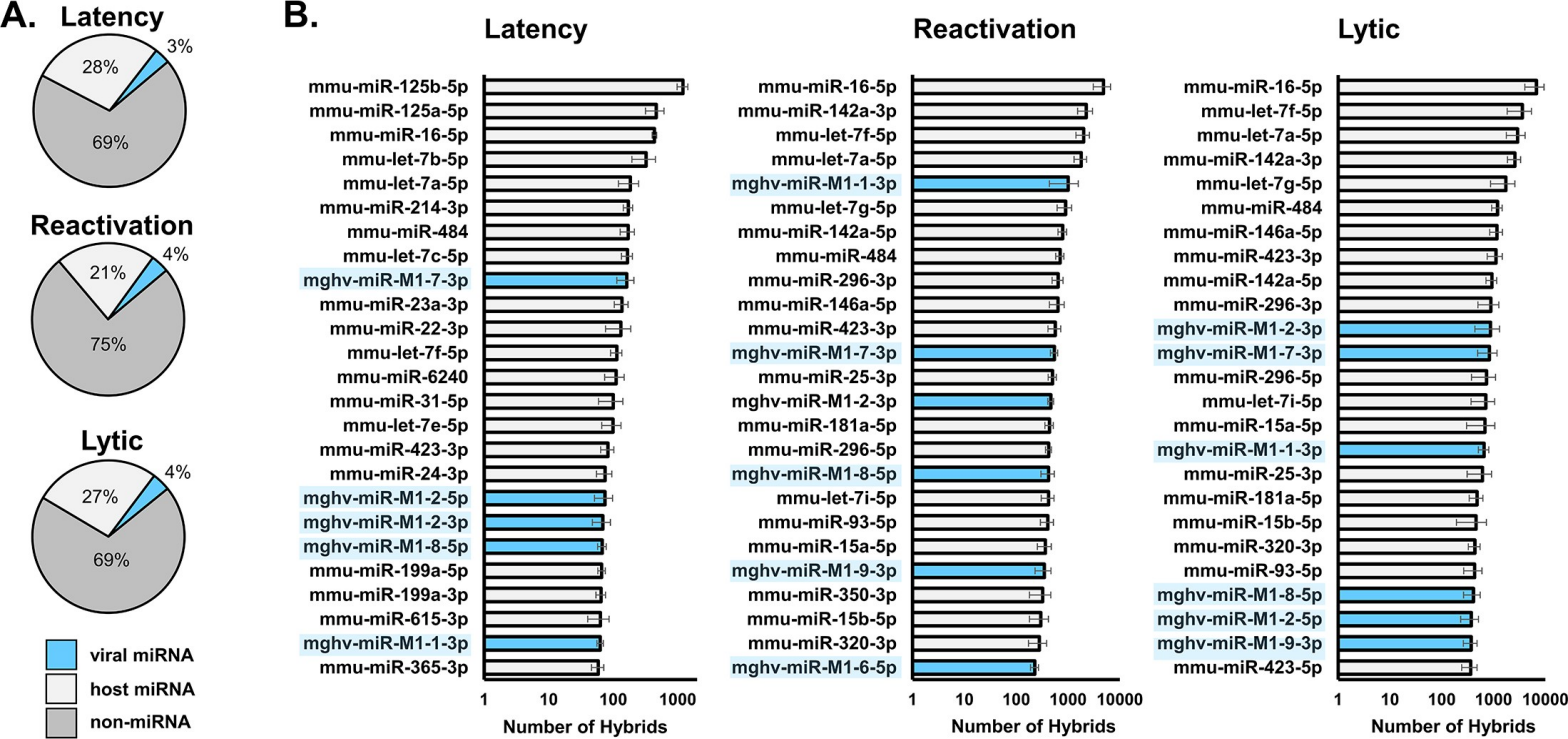
MHV68 miRNAs are among the most abundant miRNA-containing hybrids in infected cells. **A.** The percentage of rRNA-filtered RNA-RNA hybrids that contain either host or viral miRNAs during latent infection of B cells, reactivation from latency in B cells, and lytic infection in fibroblasts. **B.** The top 25 most frequent miRNAs observed in RNA-RNA hybrids during differing infection conditions. Error bars represent the standard deviation across three biological replicates. Hybrids containing MHV68 miRNAs are highlighted in blue.

Consistent with previous CLASH studies [37,45], 0.1 to 1.5% of reads were classified as RNA-RNA hybrids (**S3 Table**). Of those miRNA-mRNA hybrids that aligned to a host mRNA, between 11 and 15% carried a MHV68 miRNA across all three sample groups (**Fig 1A**). In all, we detected 844 to 1,316 MHV68 miRNA hybrids per latency replicate, 4,135 to 6,949 per reactivation replicate, and 4,331 to 7,993 per lytic replication replicate (**S3 Table**). Although the abundance of individual MHV68 miRNAs varied between sample groups, five to six MHV68 miRNAs were consistently represented among the top 25 most abundant qCLASH miRNA-containing hybrids (**Fig 1B**). MHV68 miRNAs *miR-M1-1-3p*, *-2-5p*, *-2-3p*, *-7-3*, *-8-5p* and *-9-3p* were most commonly detected during all phases of infection (**Fig 1B and Table 1**).

**Table 1:** Number of host mRNA hybrid sequences containing MHV68 miRNAs. For each hybrid containing a host mRNA, the total number of sequences containing each of the 28 potential mature MHV68 miRNAs was enumerated. For each sample type (latency, reactivation, lytic) and corresponding miRNA, numbers represent the total unique hybrids identified within the three biological replicates. miRNAs are listed in genomic order, with the corresponding TMER pri-miRNA transcript indicated.

	MHV68 miRNA	Latency	Reactivation	Lytic
**TMER1**	*mghv-miR-M1-* **1-5p**	1	2	0
	*mghv-miR-M1-* **1-3p**	354	4342	3153
	*mghv-miR-M1-* **10-5p**	1	43	67
	*mghv-miR-M1-* **10-3p**	16	213	277
**TMER2**	*mghv-miR-M1-* **2-5p**	350	750	1380
	*mghv-miR-M1-* **2-3p**	664	1840	3225
	*mghv-miR-M1-* **3-5p**	4	52	66
	*mghv-miR-M1-* **3-3p**	33	155	187
**TMER3**	*mghv-miR-M1-* ***4-5p***	3	155	170
	*mghv-miR-M1-* ***4-3p***	5	27	36
**TMER4**	*mghv-miR-M1-* ***5-5p***	67	1098	1413
	*mghv-miR-M1-* ***5-3p***	47	381	558
	*mghv-miR-M1-* ***6-5p***	165	972	835
	*mghv-miR-M1-* ***6-3p***	12	171	228
**TMER5**	*mghv-miR-M1-* ***7-5p***	138	433	598
	*mghv-miR-M1-* ***7-3p***	811	2546	3482
	*mghv-miR-M1-* ***12-5p***	3	6	1
	*mghv-miR-M1-* ***12-3p***	62	212	269
**TMER6**	*mghv-miR-M1-* ***13-5p***	2	1	1
	*mghv-miR-M1-* ***13-3p***	32	501	431
	*mghv-miR-M1-* ***8-5p***	237	1438	1430
	*mghv-miR-M1-* ***8-3p***	11	36	39
**TMER7**	*mghv-miR-M1-* ***14-5p***	7	107	103
	*mghv-miR-M1-* ***14-3p***	11	79	195
**TMER8**	*mghv-miR-M1-* ***15-5p***	0	0	0
	*mghv-miR-M1-* ***15-3p***	56	1	0
	*mghv-miR-M1-* ***9-5p***	2	1	2
	*mghv-miR-M1-* ***9-3p***	57	1353	1659

### Characteristics of virus and host miRNA binding

**Fig 2:**
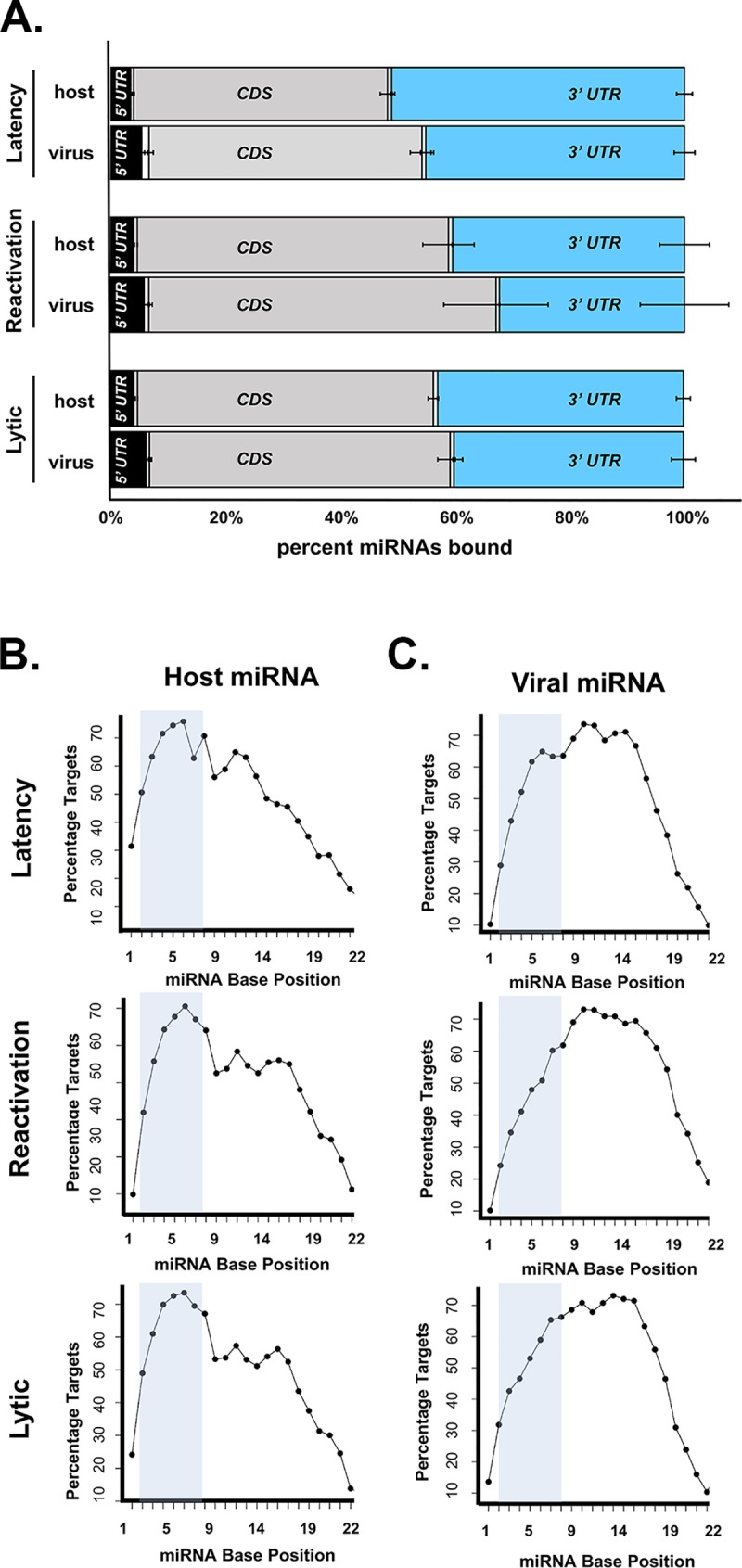
Host and MHV68 miRNAs bind frequently to the CDS or 3’ UTR of target transcripts, but MHV68 miRNAs cumulatively display a non-canonical binding profile. **A.** For all miRNA-mRNA hybrids within each infection condition, the percentage of total host or viral miRNAs bound to each region of target transcripts is indicated. Regions were categorized as 5’ UTR, 5’ UTR-CDS, CDS, CDS-3’ UTR, or 3’ UTR. Error bars represent the standard deviation across three biological replicates. The target transcript 3’ UTR (the canonical miRNA binding region) is highlighted in blue. **B, C.** For all miRNA hybrids within each infection condition, the percentage of target mRNAs that bound to individual nucleotides along the length of each miRNA was determined. Percentages were calculated for all host (B) or virus (C) miRNA-mRNA interactions within cumulative latency, reactivation and lytic datasets.

The current view of miRNAs is that they repress translation through binding interactions at the 3’ UTR of target transcripts. However, recent CLASH studies have found that numerous miRNAs target mRNA transcripts through binding to regions outside of the 3’ UTR, including the protein coding sequence [37,45,46]. To assess which domains of mRNA target transcripts were bound by Ago-associated MHV68 miRNAs, we defined whether the mRNA target sequence in individual hybrids aligned to the mRNA 5’ untranslated region (5’UTR), coding region (CDS), or 3’ untranslated region (3’UTR) (**Fig 2A**). In some case, target sequences spanned two regions and thus were classified as 5’UTR-CDS or CDS-3’UTR. Consistent with other CLASH-based findings [37], the majority of host (44 to 54%) and viral (47 to 60%) miRNAs aligned to the CDS of cognate target transcripts. The 3’ UTR was the next most frequently targeted region, accounting for 40 to 51% of host miRNA and 32 to 45% of viral miRNA binding. For both viral and host miRNAs, targeting of the 5’ UTR, 5’UTR-CDS boundary, and the CDS-3’UTR boundary cumulatively accounted for less than 10% of binding interactions. In general, viral miRNAs targeted CDS regions more frequently than host miRNAs; however, these differences were not statistically significant. Likely reflecting the distinct cellular transcriptomes during lytic infection, latency and reactivation, both host and viral miRNA targeting of specific transcript regions varied moderately among latency, reactivation, and lytic datasets, with targeting at the 3’ UTR highest during latency.

While many factors contribute to miRNA target binding and target repression, the miRNA seed sequence (defined as nucleotides 2 to 8) is conventionally thought to be one of the most important [7]. To examine the binding characteristics of host vs. viral miRNAs to their target transcripts along the length of miRNAs, we calculated the percentage of cumulative targets that were bound at each nucleotide (**Fig 2B**). As expected, the seed sequence nucleotides of host miRNAs participated in target binding at a high frequency, with lower complementarity over nucleotides 13 to 21. Interestingly, although the viral miRNAs utilized canonical seed sequence binding less frequently than host miRNAs, this was complemented by a high frequency utilization of downstream nucleotides through position 15. These characteristics were reflected in an overall rightward shift of the cumulative viral miRNA target binding curve. However, this cumulative shift reflected a strong bias toward those individual hybrid binding interactions most highly represented in the viral miRNA qCLASH binding set (**S1 to S3 Figs**). For example, within the latency group, two of the three most highly represented miRNAs, *mghv-miR-M1-7-3p* and *mghv-miR-M1-1-3p*, demonstrated unusual, non-canonical binding profiles with target mRNAs. However, other top miRNAs, including *mghv-miR-M1-2-5p* and *mghv-miR-M1-6-5p* demonstrated increased seed sequence binding, indicating that the cumulative binding profile does not reflect an overall skewing of binding by the entire viral miRNA population.

### Host mRNA targets of MHV68 miRNAs

**Fig 3:**
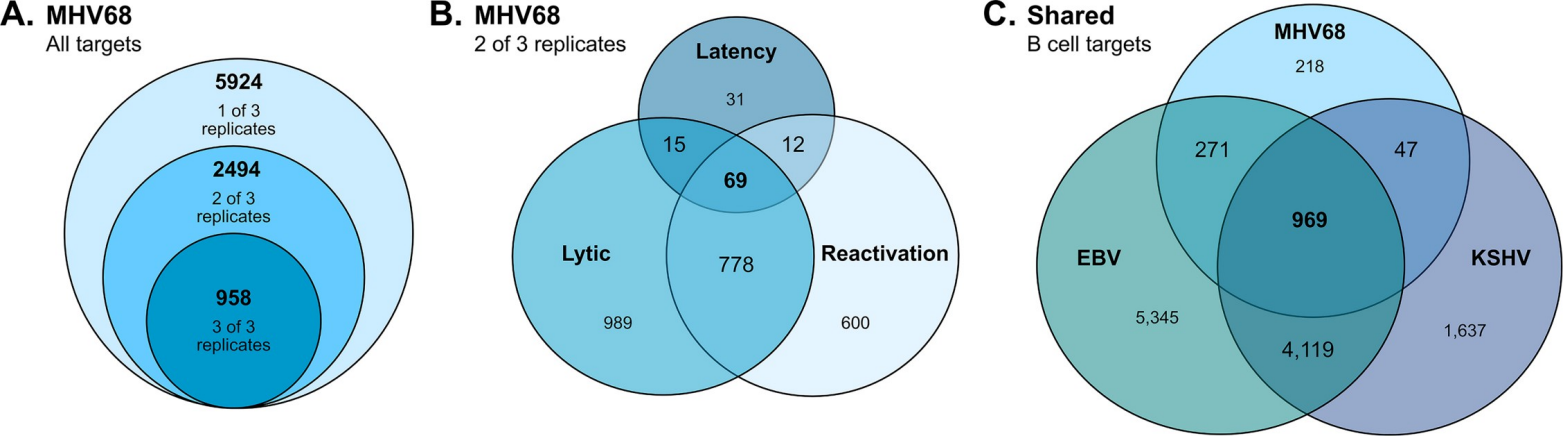
MHV68 miRNA targets extensively overlap with EBV and KSHV miRNA targets. **A.** Diagram indicates the number of unique host mRNA targets of MHV68 miRNAs identified in one, two or three biological replicates (out of three). **B.** Venn diagram indicates the number of host mRNA targets of MHV68 miRNAs (identified in at least two of three biological replicates) that are shared among lytically infected fibroblasts, latently infected B cells and/or B cells reactivated from latency. **C.** Venn diagram indicates the number of unique mRNA targets of MHV68 miRNAs identified in B cells that are shared with B cell mRNA targets of EBV miRNAs, KSHV miRNAs, or both EBV and KSHV miRNAs. MHV68 B cell targets include those identified in two of three biological replicates during latent infection or reactivation from latency. EBV and KSHV targets were identified using CLIP and have been previously published [16–21].

In total, analysis of qCLASH hybrids from latency, reactivation, and lytic datasets revealed 5,924 unique host transcripts that were cumulatively targeted by MHV68 miRNAs across three biological replicates. Of those, 2,493 and 957 were observed in two of three and three of three biological replicates, respectively (**Fig 3A**). Notably, numerous miRNA targets were shared across different phases of infection and different cell types (**Fig 3B**). For example, 76% (96 of 127) of targets detected in latently infected B cells were also detected in both reactivating B cells and lytically infected fibroblasts. Likewise, 778 targets from B cells undergoing reactivation from latency were shared with lytically infected fibroblasts.

Because gammaherpesviruses including MHV68 establish lifelong infection in B cells, we elected to focus our in-depth studies on those viral miRNA targets identified in B cell latency and/or reactivation qCLASH libraries. Of the unique host targets present in two of three biological replicates, 1,505 were observed in B cells, 57% (862 of 1,505) of which were also detected in fibroblasts (**S4 Fig**). To select for transcripts whose targeting may be conserved across gammaherpesviruses, we compared these CLASH-identified B cell targets to putative EBV and KSHV targets previously identified in CLIP datasets from B cells [16–21] (**Fig 3C**). Impressively, of the 1,505 MHV68 miRNA targets identified in B cells, 82% (1,204) were common with EBV, 68% (1,016) were common with KSHV, and 64% (969) were common with both EBV and KSHV.

To validate repression of individual host targets by MHV68 miRNAs, we selected a subset of nine transcripts, eight of which were shared with EBV and/or KSHV miRNAs (**S4 Table**). These targets include mRNAs that encode proteins relevant to chromatin remodeling, transcription, translation, and apoptosis: ARID1A, a chromatin remodeling protein [47], CTSL, a lysosomal cysteine proteinase [48], EWSR1, an RNA binding protein [49], FUS, an RNA binding protein [50], IFITM3, an interferon stimulated transmembrane protein [51], PHC3, a member of the Polycomb Group chromatin remodeling complex [52], FOXJ3, a transcription factor [53], KDM5B, a histone demethylase [54], and TRP53INP1, a proapoptotic protein [55].

**Fig 4:**
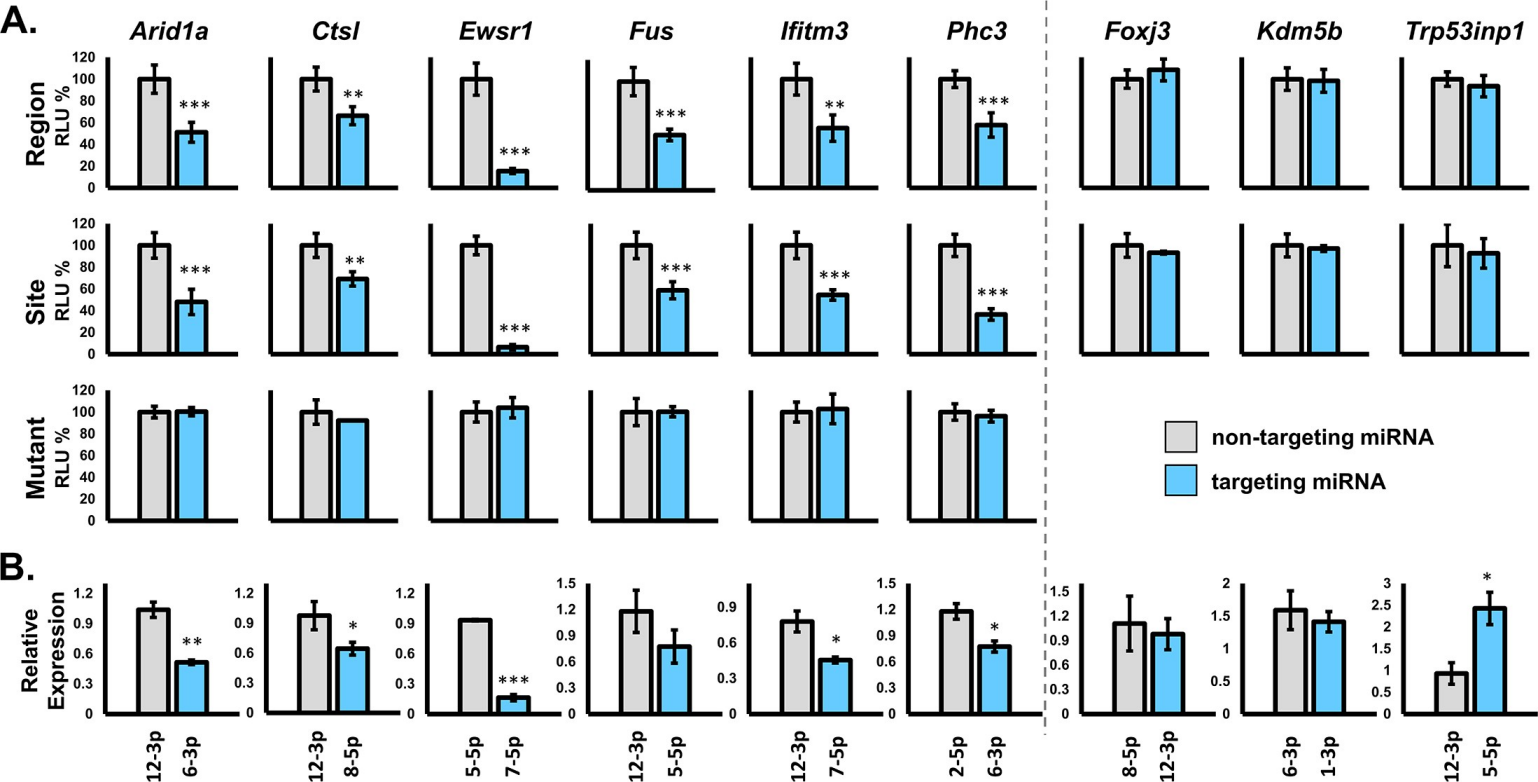
Validation of mRNA target repression by MHV68 miRNAs. **A.** Effect of qCLASH-identified targeting (blue) vs. non-targeting (gray) miRNAs on expression of lucifierase from transcripts carrying corresponding miRNA target sequences within the luciferase 3’ UTR. Target sequences are the miRNA binding site surrounded by 500–1000 nt of the native flanking sequence (Region), the miRNA binding site with 5–10 nt flanking sequence (Site), or mutated version of the miRNA binding site with 5–10 nt flanking sequence (Mutant). NIH 3T12 cells were transfected with luciferase constructs plus targeting or non-targeting control miRNA mimics, and luciferase activity was quantified 18 hours later. Luciferase activity is expressed as percent relative light units (RLU) relative to non-targeting control miRNA. Each experiment was performed in triplicate with error bars representative of standard deviation. **B.** Effect of qCLASH-identified targeting (blue) vs. non-targeting (gray) miRNAs on levels of endogenously expressed target mRNAs. NIH 3T12 cells were transfected with targeting or non-targeting control miRNA mimics, then endogenous mRNA levels were quantified 18 hours later using qRT-PCR. Y-axis values are expression relative to no miRNA controls. ***p<0.001, **p<01, *p<0.05. Each experiment was performed in triplicate with error bars representative of standard deviation.

To stringently assess MHV68 miRNA repression of these select targets we utilized complementary molecular approaches including luciferase-based 3’ UTR targeting, *in vivo* transcript stability, and protein expression. To first determine whether MHV68 miRNAs could directly repress transcripts carrying target sequences from qCLASH hybrids, we performed luciferase knockdown assays using luciferase 3’ UTR constructs carrying: (a) specific miRNA target sequences with 500 to 1000 bp native flanking sequence (“region”), (b) ~30 bp specific miRNA target site sequences (“site”), or (c) ~30 bp specific miRNA target site sequences with a 3 bp mutation in the binding sequence complementary to the miRNA seed (“mutant”). Specific target knockdown was determined for each transcript using the cognate CLASH-identified targeting MHV68 miRNA versus a control non-targeting MHV68 miRNA. For this study, we considered statistically significant reduction of luciferase activity by 30% or greater compared to control to constitute biologically relevant target repression. By this standard, transcripts carrying *Arid1a*, *Ctsl*, *Ewsr1*, *Fus*, *Ifitm3*, or *Phc3* target sequences demonstrated specific repression by the qCLASH-identified MHV68 miRNAs *mghv-miR-M1-6-3p*, *-8-5p*, *-7-5p*, and *-6-3p* respectively (**Fig 4A**). For individual targets, the level of transcript repression was similar between those transcripts carrying large target regions and those transcripts carrying minimal target sites. Moreover, in each case mutation of 3–4 bases within the target site completely ablated repression, demonstrating the specificity of miRNA targeting. As expected, not all qCLASH-identified transcripts were repressed by their targeting miRNA, as transcripts carrying *FoxJ3*, *Kdm5b*, or *Trp53inp1* target sequences were not affected by cognate miRNA binding.

To determine whether MHV68 miRNAs could also directly repress endogenously expressed mRNAs, we quantified the relative level of specific target transcripts in cells transfected with individual miRNAs (**Fig 4B**). Consistent with results from luciferase assays, the endogenous levels of *Arid1a*, *Ctsl*, *Ewsr1*, *Fus*, *Ifitm3*, or *Phc3* mRNAs were repressed by their respective qCLASH-identified miRNAs. Moreover, the endogenous levels of mRNAs *FoxJ3*, *Kdm5b*, or *Trp53inp1*, which carry target sites not repressed in luciferase assays, were not reduced in the presence of targeting miRNAs. In fact, endogenous *Trp53inp1* levels were significantly increased in the presence of targeting miRNA *mghv-miR-M1-5-5p*, implicating miRNA binding in stabilization of this transcript.

**Fig 5:**
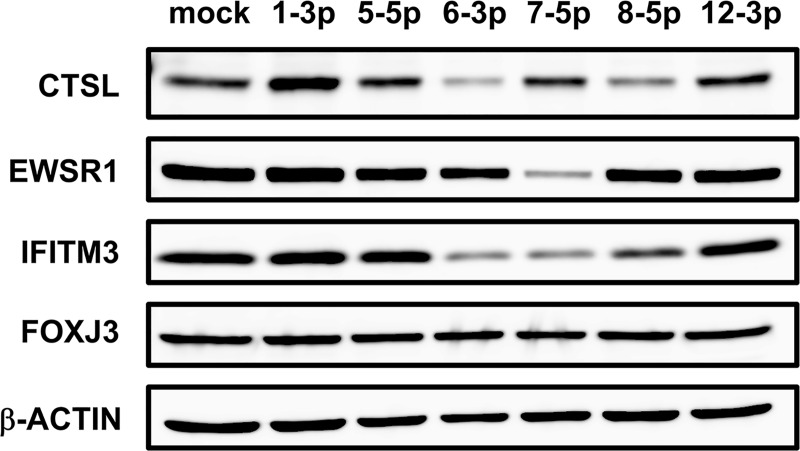
Validation of repression of target proteins by MHV68 miRNAs. Effect of qCLASH-identified targeting vs. non-targeting miRNAs on expression of proteins encoded by target transcripts. Endogenous protein levels were determined using western blot for total protein, 24 hr post-transfection. Western blots are a representative of three biological replicates. qCLASH-identified targeting miRNAs were: *miR-8-5p* for *Ctsl*, *miR-7-5p* for *Ewsr1*, *miR-7-5p* for *Ifitm3*, and *miR-12-3p* for *Foxj3*.

To determine whether reduced levels of target mRNAs correlated with reduced endogenous protein expression, we assessed protein levels of select targets in the presence of individual miRNAs (**Fig 5**). Consistent with the mRNA knockdown results, we observed reduced expression of CTSL, EWSR1, and IFITM3 proteins in the presence of their qCLASH-identified mRNA binding partners *miR-M1-8-5p*, *-7-5p* and *-7-5p*, respectively. Likewise, expression of FOXJ3 protein, whose mRNA was not repressed by *miR-M1-12-3p* binding, was not altered in the presence of *miR-M1-12-3p*. Notably, although EWSR1 protein levels were reduced by its qCLASH-identified interacting miRNA but not by other MHV68 miRNAs, the levels of CTSL and IFITM3 protein were repressed by their respective qCLASH-identified miRNAs as well as by *miR-M1-6-3p*, which was not identified by qCLASH. These observations may reflect miRNA-mRNA interactions that were not efficiently recovered by qCLASH, or miRNA repression of targets that indirectly affect expression of CTSL and IFITM3. Together, these findings validate the identification of specific miRNA-mRNA interactions by qCLASH, and demonstrate that the MHV68 TMERs yield mature miRNAs that function to suppress expression of host proteins.

**Fig 6:**
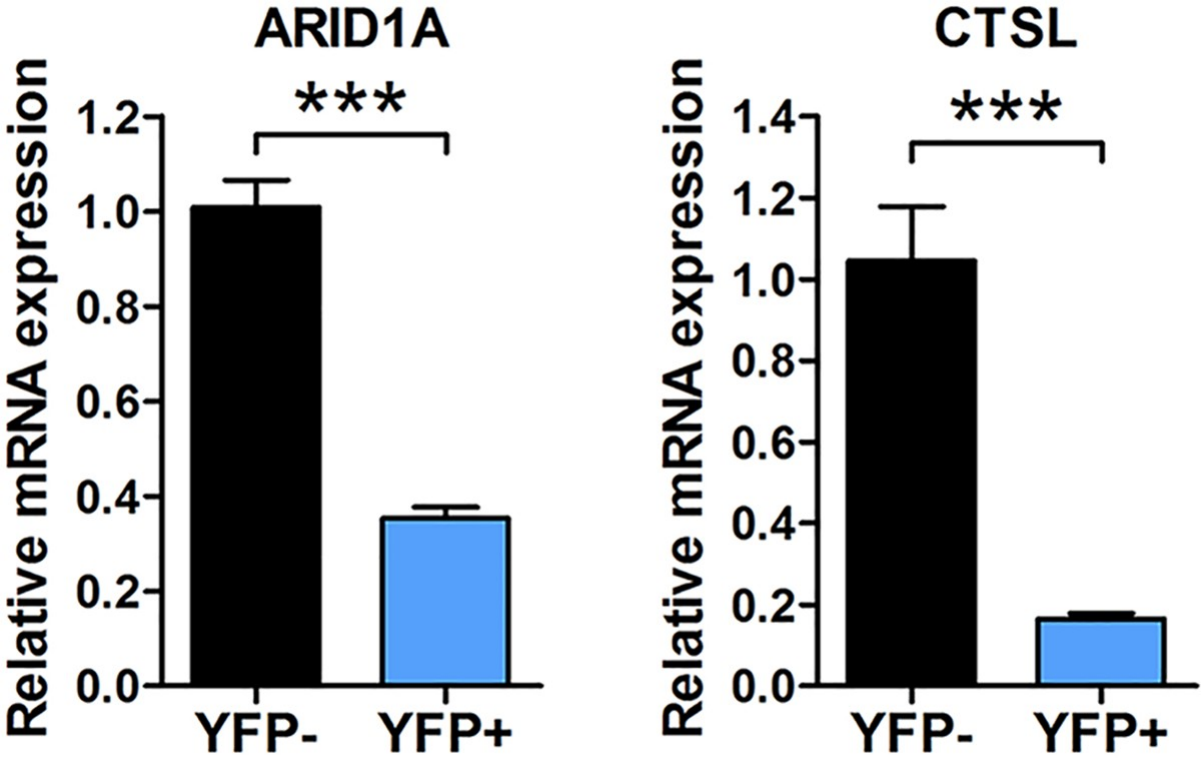
Targets of MHV68 miRNAs are repressed in vivo. The relative expression of endogenous mRNAs in non-infected versus infected B cells sorted from *in vivo* samples during chronic infection. Wild-type B6 mice were infected i.n. with 10^4^ PFU of MHV68-H2bYFP. At 16 days, splenocytes were harvested and subjected to flow cytometric sorting to isolate both non-infected B cells (CD4-CD8-CD14-CD19+YFP-) and infected B cells (CD4-CD8-CD14-CD19+YFP+). Following sorting, the transcript level of MHV68 miRNA targets *Arid1a* and *Ctsl* were determined in each sample using qRT-PCR. Values represent the mean ± SEM of three independent experiments. Significance was determined by a two-tailed, unpaired t-test. ***p< 0.001.

Like EBV and KSHV, MHV68 predominantly establishes latent infection in circulating mature B cells *in vivo* [26–28,44,56–59]. To determine whether host transcripts which were validated for repression *in vitro* were also repressed in latently infected B cells *in vivo*, we performed qRT-PCR for host transcripts of interest on pure populations of non-infected or infected B cells sorted from mice during chronic infection. Wild-type C57BL/6J mice were infected with MHV68.H2bYFP, a eYFP-marked recombinant MHV68 that is phenotypically wild-type[44]. At 16 days, splenocytes were harvested and sorted into infected (CD4-CD8-CD14-CD19+YFP+) versus non-infected (CD4-CD8-CD14-CD19+YFP-) B cell populations. Following RNA extraction, transcript levels of *Arid1a* and *Ctsl* were determined by qRT-PCR (**Fig 6**). As compared to YFP- non-infected B cells, the levels of *Arid1a* and *Ctsl* transcripts were significantly decreased in YFP+ latently infected B cells. Due to the difficulty recovering large numbers of pure populations of infected cells from *in vivo* samples, we were only able to quantify transcript levels for these two transcripts. Nevertheless, these data clearly demonstrate that these two qCLASH-identified targets of MHV68 miRNAs were repressed in infected cells *in vivo*, strongly supporting the concept that a large subset of the host targets identified in this study are likely *bona fide* targets repressed by MHV68 miRNAs *in vivo*.

### Host pathways targeted by MHV68 miRNAs

**Fig 7:**
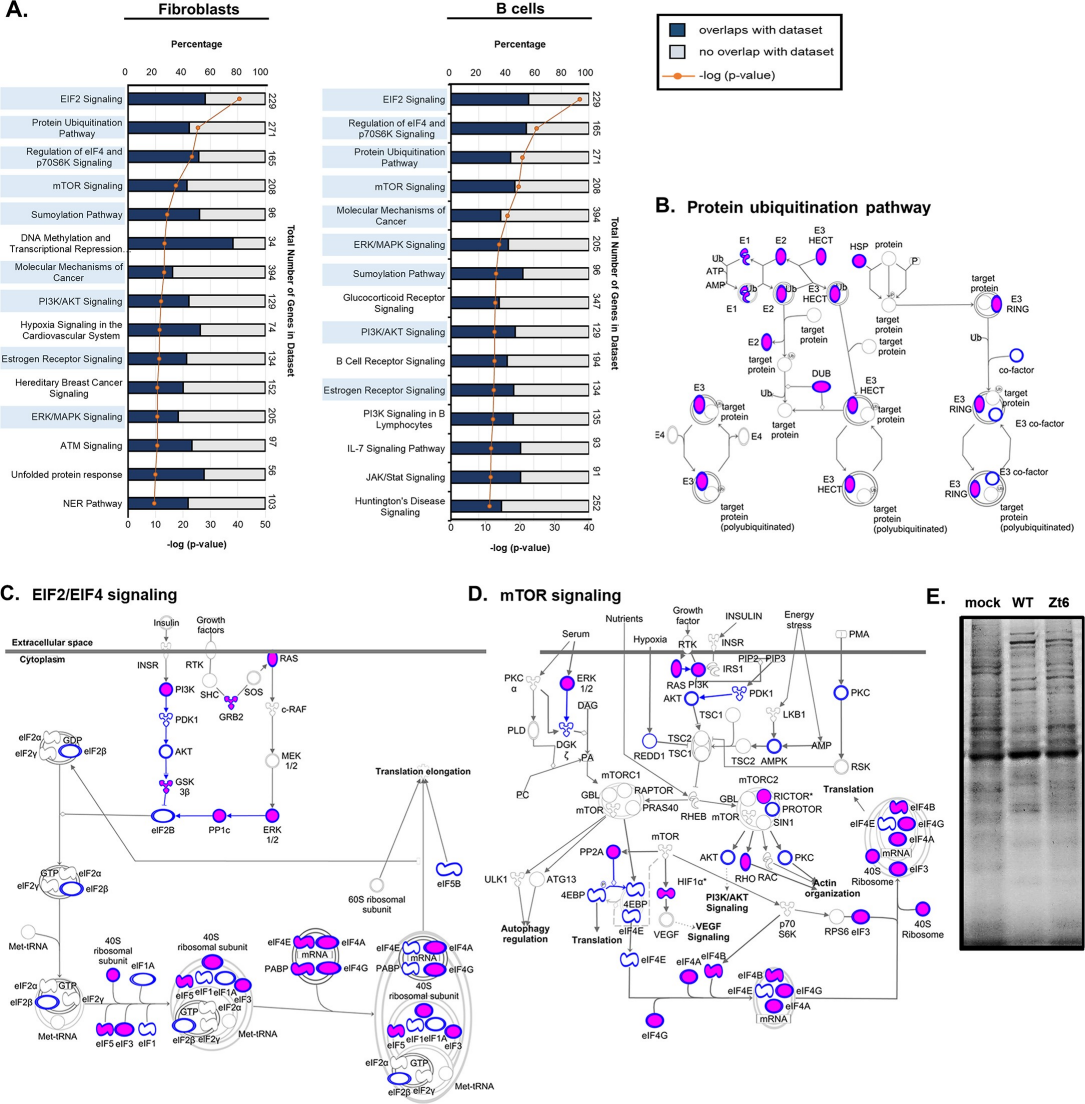
Canonical signaling pathways targeted by MHV68 miRNAs in fibroblasts vs. B cells, and conservation of MHV68, EBV and KSHV B cell-derived miRNA targets. **A.** The top 15 canonical pathways enriched for targets of MHV68 miRNAs in either fibroblasts (left panel) or B cells (right panel). Bar graphs correspond with the top X-axes, and depict the percent of target transcripts that overlap (blue) with each dataset containing defined members of individual pathways. The total number of genes in each pathway dataset are indicated on the right. The line graph overlays indicate the -log p value for target enrichment within each dataset, and points correspond with the bottom X-axes. Ingenuity Pathway Analysis was performed using cumulative mRNA targets from fibroblasts (lytic infection) or B cells (latent infection and reactivation) that were identified in at least two of three biological replicates for specified infection conditions. Labels for pathways common to both fibroblasts and B cells are highlighted in light blue. **B, C, D.** Conservation of MHV68, EBV and KSHV B cell-derived miRNA targets in host signaling pathways defined by IPA. Diagrams show the canonical host (A) protein ubiquitination, (B) eIF2 and eIF4 signaling, and (C) mTOR signaling pathways. Blue outline overlays of individual pathway components indicate targets of MHV68 miRNAs identified by qCLASH in B cells. Magenta fill overlays of individual pathway components indicate MHV68 B cell-derived miRNA targets shared with B cell-derived targets of EBV, KSHV or both EBV and KSHV miRNAs.

To determine if there were major cellular pathways which were highly targeted by MHV68 miRNAs, pathway enrichment analysis was performed using the 1,851 and 1,505 genes which were identified in at least two of three qCLASH biological replicates for fibroblasts and B cells, respectively (S4 Fig). Ingenuity Pathway Analysis (IPA) defined significant gene enrichment in numerous canonical cellular pathways with known relevance to cancer biology, including more than 35% of the 394 host genes categorized within the molecular mechanisms of cancer pathway (**Fig 7A, S5 Table**). Notably, eIF2 signaling, protein ubiquitination, regulation of eIF4 and p70S60K signaling, and mTOR signaling, were the four most highly enriched pathways for both fibroblasts and B cells. Moreover, nine of the top twelve pathways in fibroblasts were represented within the top eleven pathways in B cells, demonstrating conserved pathway targeting between cell types. Despite the significant overlap between target sets, analysis of individual cell types nevertheless revealed enhanced enrichment of some pathways in one cell type versus the other. For example, using the targets identified in B cells revealed a high level of enrichment in pathways highly active or exclusive to lymphocytes, including B cell receptor signaling, B cell PI3K signaling, IL-7 signaling pathway, and JAK/Stat signaling. Likewise, the DNA methylation and transcriptional repression pathway was very highly enriched in fibroblasts but not B cells, perhaps reflecting the need for the virus to counteract host transcriptional repression mechanisms during lytic replication.

One of the most notable observations for both cell types was the high representation of pathways directly involved in protein translation or protein modification within the target sets demonstrating the most significant enrichment (Fig 7A, S5 Table). This group includes eIF2 signaling, eIF4 and p70S6K signaling, mTOR signaling, protein ubiquitination pathway, and sumoylation pathway. Interestingly, comparison of MHV68 miRNA targets derived from B cells with EBV and KSHV miRNA targets previously identified in B cells [16–21], revealed significant conservation of targeting within individual pathways. In particular, a high percentage of the MHV68 B cell-derived miRNA targets integral to translation and protein modification pathways were shared with EBV and/or KSHV miRNA B cell targets. These include the protein ubiquitination pathway (**Fig 7B**), eIF2/eIF4 signaling (**Fig 7C**) and mTOR signaling (**Fig 7D**). For example, numerous critical components of the eIF4 complex, including eIF4A, eIF4B, eIF4E, eIF4G, eIF3 and eIF5, were targeted by MHV68 and at least one of the human viruses. These findings clearly demonstrate conserved targeting of protein translation and modification pathways by gammaherpesvirus miRNAs.

To determine if there were functional consequences for MHV68 miRNA targeting of pathways involved in host translation, we assessed global translation in cells infected with wild-type MHV68 or with MHV68.Zt6, a previously published MHV68 mutant deficient in the expression of all 14 pre-miRNAs [34]. After 10 hours of infection, newly synthesized proteins were labeled through ^35^S-methionine incorporation, and cell lysates where then subjected to SDS-PAGE gel electrophoresis. As expected, imaging of radiolabeled proteins revealed a reduction in both individual resolved bands and unresolved background bands in wild-type MHV68-infected cells as compared to mock-infected cells (**Fig 7E**), indicating that infection resulted in a significant reduction in the translation of new proteins. Notably though, translation was nearly restored to a level equivalent to mock in cells infected with the miRNA-deficient virus MHV68.Zt6. These findings demonstrate that viral miRNAs contribute to the global reduction in translation observed following MHV68 infection.

## Discussion

miRNAs are critical regulators of gene expression which function by binding to cognate sequences of a mRNA target in the context of the Ago-associated RNA silencing complex. Viral miRNAs are thought to benefit distinct phases of virus infection through repression of specific host mRNA targets and through combined repression of multiple components of host cell pathways. Numerous targets of EBV and KSHV miRNAs have been identified; however, their functions *in vivo* infection remain poorly understood. Although MHV68 infection of mice represents an outstanding system to define the *in vivo* function of conserved gammaherpesvirus miRNAs, to date, specific targets of MHV68 miRNAs have not been identified. Here, we applied the qCLASH technique to identify mRNA targets of MHV68 miRNAs during latent infection of B cells, reactivation of B cells, and lytic infection of fibroblasts. Using this approach, we defined thousands of high-confidence mRNA targets of MHV68 miRNAs, and validated these results by demonstrating miRNA-mediated repression of select targets at both the transcript and protein level. Importantly, 86% of qCLASH-identified mRNA targets in B cells were shared with published EBV and/or KSHV targets in B cells [16–21], and 64% were shared among all three viruses, demonstrating conserved strategies for repression of host proteins among gammaherpesviruses.

### qCLASH miRNA-mRNA hybrid data set and validations

In qCLASH experiments presented here, we identified 2,493 unique host transcripts that were targeted by MHV68 miRNAs in at least two of three biological replicates. Likely owing to the very low efficiency of the ligation of Ago-associated RNAs, the recovery of RNA-RNA hybrids was very low (S3 Table). This was expected and highly consistent with previously published CLASH-based studies [37,45]. Among Ago-associated RNA-RNA hybrids recovered, approximately 41% contained either host or viral miRNAs. Of these, 13% contained MHV68 miRNAs. Although the total number of viral miRNA hybrids recovered during latency was 3- to 4-fold lower than the numbers recovered during lytic infection and reactivation, this was a reflection of the total number of reads, as viral miRNAs still represented 13% of the total miRNA hybrids recovered during latency. Moreover, 76% (96 of 127) of hybrids detected during latency were also found in lytic and/or reactivation samples (Fig 3B).

Despite the low efficiency of ligation, it is anticipated that the vast majority of RNA hybrids obtained through CLASH-based approaches represent legitimate miRNA targeting of mRNAs. This is due to the stringency of the procedure, in which (a) Ago-associated RNA hybrids are recovered only after Ago precipitation, RNase treatment, and ligation of protected RNAs, and (b) valid miRNA-mRNA hybrids are further identified through computational assessment of sequence complementarity within the short read. Nevertheless, *bona fide* miRNA-mRNA binding associations do not necessarily equate with reduction of target transcript and/or protein. Thus to validate repression of individual host targets of MHV68 miRNAs, we performed extensive molecular assessment (Fig 4) of nine select targets in B cells that were targets shared with EBV and/or KSHV miRNAs. Of these, six mRNA targets were significantly repressed by their cognate miRNA binding partner, and three were not altered, equating to a 67% rate of repression for miRNA-mRNA interactions in this limited sample set. Importantly, the inclusion of entire mRNA target regions versus specific mRNA target site resulted in equivalent repression, demonstrating the specificity of target site recovery through qCLASH hybrid sequencing. It is not surprising that some miRNAs did not reduce mRNA or protein levels, as it has been previously noted across species and across target identification approaches that a sizeable percentage of miRNA binding interactions do not lead to target repression [7]. The findings presented here demonstrate the utility of the qCLASH approach, and strongly suggest that the majority of qCLASH-identified hybrids represent legitimate Ago-associated binding interactions between MHV68 miRNAs and their specific mRNA targets.

Despite the legitimacy of the qCLASH approach, based on the data presented here it also reasonable to conclude that the procedure does not capture all miRNA-mRNA interactions. For example, in agreement with data presented here, *miR-M1-1-3p* and *miR-M1-8-5p* are among the most highly expressed MHV68 miRNAs during lytic and latent infection [34]. In contrast, while *miR-M1-15-5p* is clearly expressed in infected cells [34], we detected no *miR-M1-15-5p* hybrids. Further, our finding that CTSL and IFITM3 were repressed by *miR-M1-6-3p*, but that this miRNA was not recovered as a qCLASH hybrid with CTSL or IFITM3 mRNAs, may suggest that these specific miRNA-mRNA interactions were not recovered due to low abundance or poor RNA ligation efficiency. Alternatively, it is possible that CTSL and IFITM3 are not direct *miR-M1-6-3p* targets, and that instead this miRNA represses a particular gene or set of genes responsible for the expression or stability of CTSL or IFITM3. Likewise, some miRNAs frequently recovered in qCLASH have been observed to be expressed at low levels in infected, strongly suggesting that miRNA expression profiles are not predictive of Ago-mediated target repression.

It is important to note that we have limited this study to the specific examination of MHV68 miRNA targeting of host mRNAs. However, it is well-established that gammaherpesviruses not only utilize their miRNAs to regulate the expression of host genes but also use their miRNAs in order to target and regulate the expression of their own genes to regulate their lifecycle and virulence. For example, it has been demonstrated that viral miRNAs of some herpesviruses directly target lytic genes to suppress lytic reactivation and maintain latency. For example, EBV miR-BART20-5p targets the 3’ UTRs of the transcripts that encode the key latent to lytic switch proteins Rta and Zta [60]. Similarly, KSHV miR-K12-7 and -9 target the Rta transcript to maintain latency and prevent reactivation [61–64]. While the viral targets of MHV68 miRNAs have not been discussed here, future studies will investigate the viral targets of MHV68 miRNAs and how this targeting plays a role in the maintenance of latency.

### Properties of MHV68 miRNA binding interactions

The current view for miRNA function incorporates a set of well-accepted rules [7,65] for miRNA-mRNA binding interactions: (i) binding does not have to be perfectly complementary across the entire length of the miRNA, (ii) binding is largely dominated by complementarity within nucleotides 2–7 of the miRNA, a region that is defined as the miRNA seed sequence, (iii) base pairing outside of the seed sequence does not influence miRNA function, but stabilizes miRNA-mRNA binding, (iv) miRNA binding generally occurs in the 3’ UTR of target transcripts within relatively unstructured regions. Thus, while base pairing within the seed sequence is thought to be the most important determinant of miRNA targeting and function, base pairing outside of the seed sequence at the 3’ end of the miRNA, is thought to play a secondary role by stabilizing the miRNA-target transcript binding and increasing target specificity [7,65].

However, the recent application of RNA-RNA ligation-based approaches is challenging the rigidity of these rules. For example, a recent study demonstrated preferential miRNA binding of both host and viral miRNAs to the coding region (CDS) of target transcripts as comparted to the 3’ UTR [37]. Consistent with these findings, here we present data demonstrating that although a large proportion of virus and host miRNAs do bind the 3’ UTR of target transcripts, a majority of miRNAs instead bind to the coding region (CDS) of the mRNA. Interestingly, while we observed canonical seed paring with less 3’ binding in host miRNA-host mRNA hybrids, viral miRNA-host mRNA hybrids displayed decreased 5’ complementarity and a reciprocally increased 3’ complementarity. The genesis of this altered miRNA binding is unclear, but is not simply a reflection of skewing for an individual miRNA or miRNA-mRNA hybrid: Only one of the top six most highly represented miRNAs in each of the three infection data sets demonstrates a canonical target complementation with high seed sequence complementarity and lower 3’ complementarity (S1–S3 Figs). Nevertheless, these miRNAs are fully functional, as *miR-M1-8-5p* and *miR-M1-5-5p* both repressed target mRNAs in our validation studies. Interestingly, it is has been very recently reported that some miRNAs may repress translation by targeting specific recognition elements within CDS sequences, and that these interactions typically require extensive base pairing in the 3’ portion of the miRNA [66]. It is also conceivable that at least some of these binding profiles could be explained in part by competing endogenous RNAs, which have been postulated to regulate the stability of some miRNA targets through miRNA sponging; however the high frequency of repressive miRNA-mRNA interactions in our validation studies argues against this possibility.

### Pathways targeted by MHV68 miRNAs

Though individual miRNA molecules typically only affect a handful of individual mRNA targets, the synergistic actions of multiple miRNAs can substantially influence entire signaling pathways. Here we analyzed the potential influence of MHV68 miRNAs on host processes by examining the enrichment for qCLASH-identified mRNA targets in IPA-defined pathways. Transcripts targeted by MHV68 miRNAs were involved in a wide of array of key cellular pathways, including those associated with translation, protein modification, B cell signaling, and DNA damage.

In particular, pathways involved in protein translation were among the most frequently targeted, with three of the top four most significant pathways in both B cells and fibroblasts directly influencing or participating in translation: eIF2 signaling, eIF4 and p70S6K signaling, and mTOR signaling. Importantly, numerous components of these same translation and protein modification pathways were also targeted by EBV and/or KSHV miRNAs (Fig 7C and 7D), revealing a conserved strategy among gammaherpesvirus miRNAs for targeting these pathways. The specific functional consequences of miRNA targeting of translation pathway components are not yet understood. However, in support of a possible contribution of MHV68 miRNA-mediated repression of translation, the reduced level of global translation observed in cells infected with wild-type MHV68 was largely ablated in cells infected with an MHV68 mutant deficient in miRNA expression. This is perhaps surprising considering that numerous viruses, including EBV, KSHV and MHV68, are known to induce shutoff of host protein synthesis through the use of a virus-encoded alkaline exonuclease [67–69]. However, it is plausible that, in the context of virus infection, gammaherpesvirus miRNA repression of translation factors is a critical step in initiation of exonuclease-mediated expression or activity, or that regulation of translation factors is crucial for tipping the counterpoise between host protein loss and new protein synthesis in favor of the viral exonuclease. Alternatively, it is possible that viral miRNA regulation of other host or viral factors are required for exonuclease activity, or to counteract repressors of exonuclease expression. Nevertheless, the finding that EBV, KSHV, and MHV68 miRNAs all target numerous factors within pathways important for regulation of translation strongly implies that this activity may be conserved among gammaherpesviruses.

The comparison of the top fifteen pathways most significantly targeted in fibroblasts versus B cells revealed both substantial commonality at the top of the lists, and substantial differences at the bottom of the lists. In addition to the common targeting of pathways involved in host translation between cell types, protein ubiquitination and sumoylation pathways featured highly in both lists. This is perhaps not surprising, considering the wide number of viruses that are known to suppress host post-translational modification processes as a means to counteract obstacles imposed by the host cell [70,71]. In contrast, the observation of estrogen receptor signaling as a significantly enriched pathway in both cell types was perhaps surprising. However, estrogen receptor signaling has recently gained increasing attention for its functions in regulation of gene expression, particularly with regard to its involvement in regulation of epigenetic modifiers [72]. Interestingly, estrogen receptor signaling has previously been connected to regulation of B cell activation and B cell development [73], and accumulating evidence has implicated a functional role for estrogen receptor signaling in multiple B cell malignancies [74]. The common miRNA targeting of estrogen receptor signaling in both fibroblasts and B cells strongly suggests that this pathway may play a more integral role in gammaherpesvirus infection than previously appreciated.

As may be expected, pathways specific to, or particularly important for, lymphocytes were uniquely enriched in miRNA target sets identified in B cells. For example, B cell receptor signaling, IL-7 signaling, and JAK/Stat signaling were among the six pathways enriched in B cells but not fibroblasts. Repression of selective host B cell activation signaling components would be consistent with the need for these viruses, as an integral part of their lifestyles, to directly manipulate normal B cell biology in favor of virus-encoded signaling cues [75,76]. Considering the need of these viruses to counter host defenses aimed at shutting down viral gene expression, the finding that DNA methylation and transcriptional repression was among the top 6 most significantly enriched pathways in fibroblasts was not surprising. The fact that targeting of this pathway was prominent in fibroblasts but not B cells was unexpected; however, this finding may reflect the specific need for the virus to overcome repressive host mechanisms during robust lytic replication.

The findings presented here reveal a wide range of host pathways targeted by MHV68 miRNAs, and suggest that cumulative targeting of factors within these pathways may have important functional outcomes during particular stages of infection. However, it should be noted that more than 30% of qCLASH-identified miRNA-mRNA interactions did not result in repression of the target mRNA or protein in the validation of a limited number of targets presented here. This observation serves to remind that the binding of a specific miRNA to a specific mRNA target does not always result in inhibition of that target; indeed in some scenarios binding can result in target stabilization. That said, the majority of qCLASH-identified targets were significantly repressed in validation assays, implying that most pathways enriched in MHV68 miRNA target sets will likely demonstrate some level of functional repression. Nevertheless, significant efforts to validate both the targeting of individual pathway components and the effects on entire pathways will be needed in order to make more definitive conclusions about the consequences of these myriad interactions.

### Summary and future directions

The use of RNA-RNA ligation approaches is quickly transitioning the miRNA target identification field from a bioinformatics-driven narrowing of potential mRNA targets which fit within defined parameters, to the outright discovery of *bona fide* mRNA targets that in many cases defy expected parameters. Here, we have defined a set of miRNA-mRNA binding interactions, a large proportion of which result in target repression, but that do not necessarily target the mRNA 3’ UTR and do not always bind through robust seed sequence-based complementarity. The reasons for these deviations from “normal” are unknown; however, the consequences of miRNA binding appear to be largely the same as would be expected, with repression of target mRNAs. Moreover, as combined miRNA targeting of pathways associated with translation signaling resulted in overall lower protein synthesis, it is plausible that combined target repression may result in modulation of numerous other crucial cell signaling pathways. The analysis presented here will allow for continued investigation of the functional consequences of targeting of these pathways and the role that these miRNA-mRNA interactions play in the biology of gammaherpesviruses. In particular, detailed *in vivo* analyses of the shared targets of MHV68, EBV and KSHV targets should shed important new light on the consequences of the conserved repression of host pathways during chronic gammaherpesvirus infection and pathogenesis.

## Supporting information

S1 Fig Individual host mRNA binding profiles of the six most highly represented MHV68 miRNAs during latency. For all MHV68 miRNA-host mRNA hybrids identified during latent infection of B cells, the percentage of target mRNAs that bound to individual nucleotides along the length of each individual miRNA was determined. For each miRNA, n indicates the number of miRNA-mRNA hybrids identified within that infection condition.
(PDF)

S2 Fig Individual host mRNA binding profiles of the six most highly represented MHV68 miRNAs during reactivation. For all MHV68 miRNA-host mRNA hybrids identified during reactivation from latency in B cells, the percentage of target mRNAs that bound to individual nucleotides along the length of each individual miRNA was determined. For each miRNA, n indicates the number of miRNA-mRNA hybrids identified within that infection condition.
(PDF)

S3 Fig Individual host mRNA binding profiles of the six most highly represented MHV68 miRNAs during lytic infection. For all MHV68 miRNA-host mRNA hybrids identified during lytic infection of fibroblasts, the percentage of target mRNAs that bound to individual nucleotides along the length of each individual miRNA was determined. For each miRNA, n indicates the number of miRNA-mRNA hybrids identified within that infection condition.
(PDF)

S4 Fig MHV68 miRNA target overlap between B cells and fibroblasts. Venn diagram indicates the number of host mRNA targets of MHV68 miRNAs (identified in at least two of three biological replicates) that are shared between fibroblasts (lytic infection) and B cells (latent infection and reactivation).
(PDF)

S5 Fig Canonical signaling pathways targeted by all MHV68 miRNAs. The top 15 canonical pathways enriched for targets of all MHV68 miRNAs (combined from fibroblast and B cell data sets). The bar graph corresponds with the top X-axis, and depicts the percent of target transcripts that overlap (blue) with each dataset containing defined members of individual pathways. The total number of genes in each pathway dataset are indicated on the right. The line graph overlay indicates the -log p value for target enrichment within each dataset, and points correspond with the bottom X-axis. Ingenuity Pathway Analysis was performed using cumulative mRNA targets from latent, reactivation and lytic infection samples that were identified in at least two of three biological replicates for at least one infection condition.
(PDF)

S1 Table Sequences of primers used for luciferase vector cloning. Primers used for amplification of target regions, target sites, or mutated target sites are indicated.
(PDF)

S2 Table Sequences of primers used for qRT-PCR analyses. Primers used for PCR amplification of target transcripts are indicated.
(PDF)

S3 Table Number of reads and hybrids identified within biological replicates and individual infection groups. Numbers indicate the total reads or hybrids recovered in each qCLASH replicate for each sample group (lytic, reactivation or latency). Total number of virus plus host miRNA-containing hybrids (“total miRNA hybrids”) is indicated and reflects a proportion of all total RNA-RNA hybrids (“total number of hybrids”) obtained in Hyb output.
(PDF)

S4 Table Binding characteristics of MHV68 miRNA-host mRNA hybrids selected for validation. For each host mRNA selected for validation, columns indicate: the miRNA contained within the hybrid, the number of different binding sites for the miRNA located within the transcript, the total number of interactions observed for this mRNA-miRNA combination, the mRNA transcript region targeted by miRNA binding, the complementarity of binding within the miRNA seed sequence (nt 2–8), the extent of complementarity downstream of the seed sequence, and whether or not the target is found within previously published EBV and KSHV CLIP data sets. In addition, a visual representation of the miRNA-mRNA interaction is presented.
(PDF)

S5 Table MHV68 miRNA targets enriched in canonical host signaling pathways. Targets of MHV68 miRNAs found to be enriched within the top 15 canonical host signaling pathways, as identified by Ingenuity Pathway Analysis.
(PDF)
