# Supplementary figures and images for: Identification of murine gammaherpesvirus 68 miRNA-mRNA hybrids reveals miRNA target conservation among gammaherpesviruses including host translation and protein modification machinery
Source: PLoS Pathog. 2019 Aug 8;15(8):e1007843. doi: 10.1371/journal.ppat.1007843 (PMC6687095; doi:10.1371/journal.ppat.1007843)

Figure S1

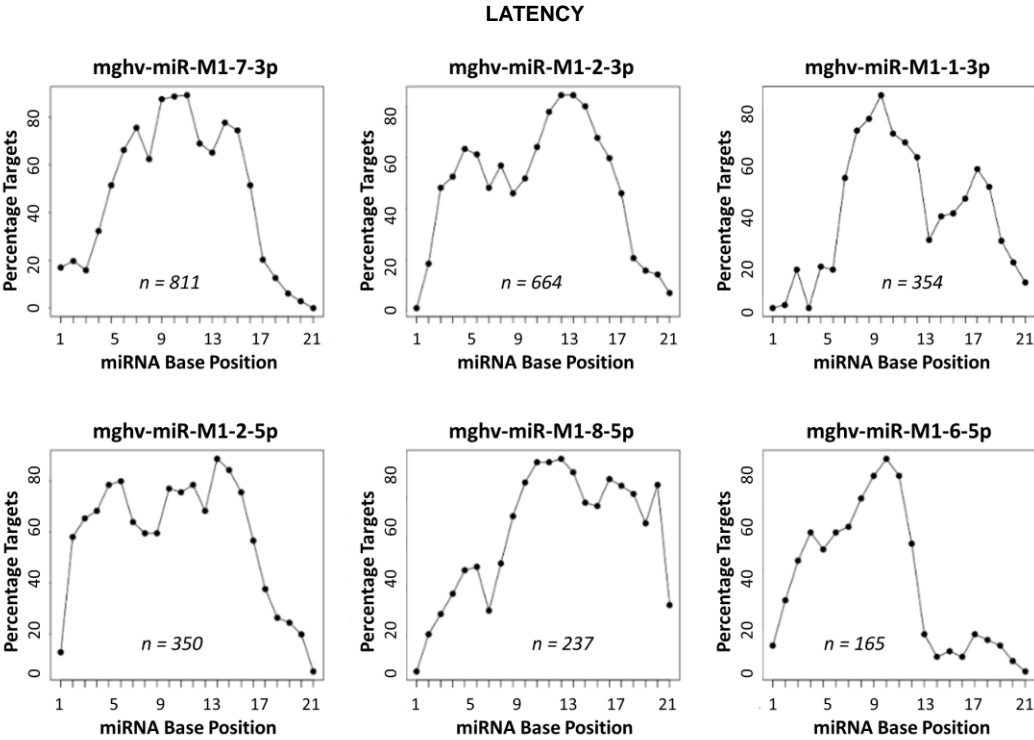

Supplement: S1 Fig — For all MHV68 miRNA-host mRNA hybrids identified during latent infection of B cells, the percentage of target mRNAs that bound to individual nucleotides along the length of each individual miRNA was determined. For each miRNA, n indicates the number of miRNA-mRNA hybrids identified within that infection condition. (PDF) [file ppat.1007843.s001.pdf]

Figure S2

REACTIVATION

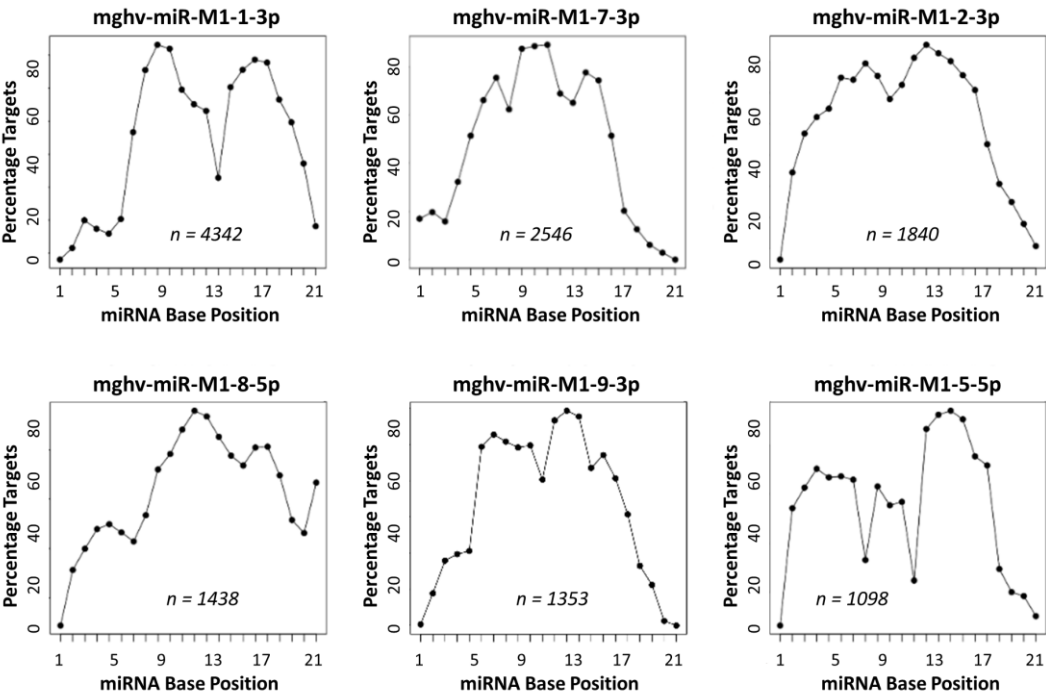

Supplement: S2 Fig — For all MHV68 miRNA-host mRNA hybrids identified during reactivation from latency in B cells, the percentage of target mRNAs that bound to individual nucleotides along the length of each individual miRNA was determined. For each miRNA, n indicates the number of miRNA-mRNA hybrids identified within that infection condition. (PDF) [file ppat.1007843.s002.pdf]

Figure S3

LYTIC

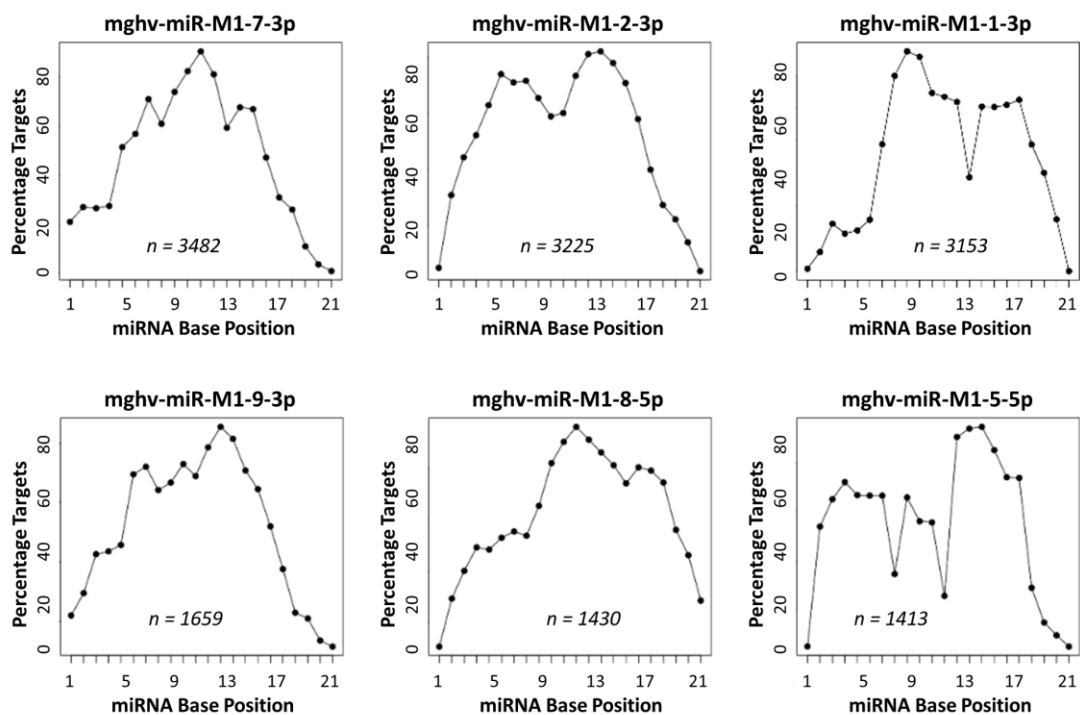

Supplement: S3 Fig — For all MHV68 miRNA-host mRNA hybrids identified during lytic infection of fibroblasts, the percentage of target mRNAs that bound to individual nucleotides along the length of each individual miRNA was determined. For each miRNA, n indicates the number of miRNA-mRNA hybrids identified within that infection condition. (PDF) [file ppat.1007843.s003.pdf]

**Figure S4**

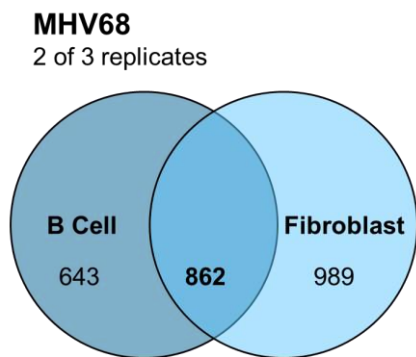

Supplement: S4 Fig — Venn diagram indicates the number of host mRNA targets of MHV68 miRNAs (identified in at least two of three biological replicates) that are shared between fibroblasts (lytic infection) and B cells (latent infection and reactivation). (PDF) [file ppat.1007843.s004.pdf]

**Figure S5**

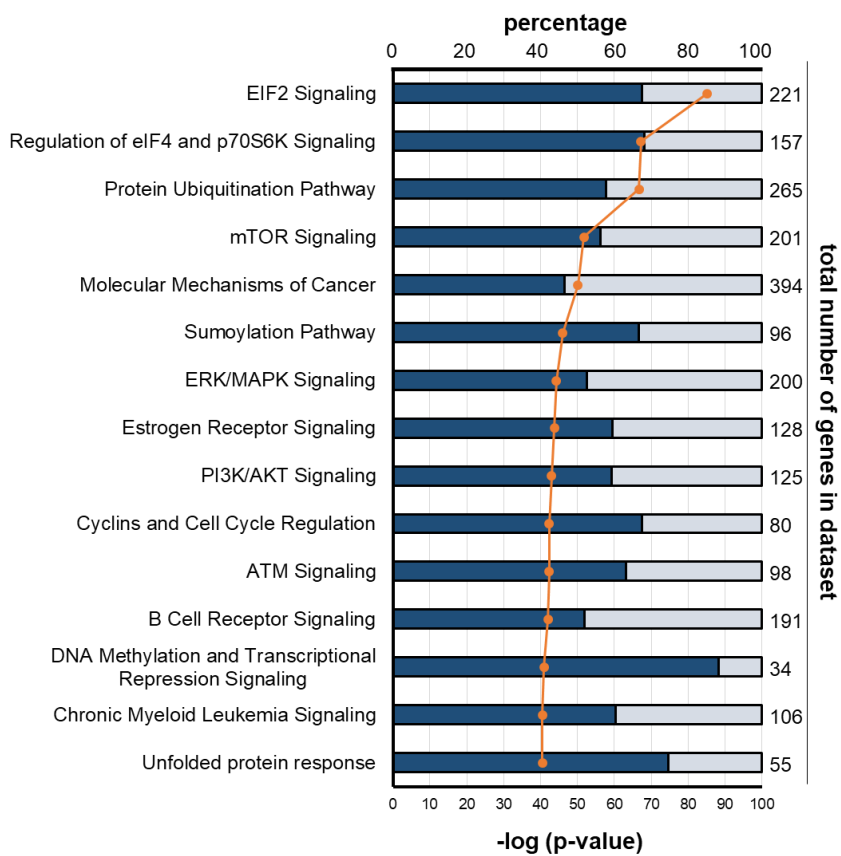

Supplement: S5 Fig — The top 15 canonical pathways enriched for targets of all MHV68 miRNAs (combined from fibroblast and B cell data sets). The bar graph corresponds with the top X-axis, and depicts the percent of target transcripts that overlap (blue) with each dataset containing defined members of individual pathways. The total number of genes in each pathway dataset are indicated on the right. The line graph overlay indicates the -log p value for target enrichment within each dataset, and points correspond with the bottom X-axis. Ingenuity Pathway Analysis was performed using cumulative mRNA targets from latent, reactivation and lytic infection samples that were identified in at least two of three biological replicates for at least one infection condition. (PDF) [file ppat.1007843.s005.pdf]
