# Supplementary material for: Identification of murine gammaherpesvirus 68 miRNA-mRNA hybrids reveals miRNA target conservation among gammaherpesviruses including host translation and protein modification machinery
Source: PLoS Pathog. 2019 Aug 8;15(8):e1007843. doi: 10.1371/journal.ppat.1007843 (PMC6687095; doi:10.1371/journal.ppat.1007843)
Supplement: S1 Table — Primers used for amplification of target regions, target sites, or mutated target sites are indicated. (PDF) [file ppat.1007843.s006.pdf]

Table S1

Sequences of primers used for luciferase vector cloning.

Target	Sense	Antisense
**Arid1a Target Region**	5'-ATCGCCGTGTAATTCTAGTTGTTTATGCAGAATCCACCCTTTGA-3'	5'-AGACTCGAGGCTAGCGAGCTCGTTTCGTTGTAGGTTCACACAGGATA-3'
**Arid1a Target Site**	5'-**C**TATGCAAAACCACCTCAGAATCCAGTTTACCCTGTGCTG**T**-3'	5'-**CTAGA**CAGCACAGGGTAAACTGGATTCTGAGGTGGTTTTGCATA**GAGCT**-3'
**Arid1a Mutant Target Site**	5'-**C**TATGCAAAACCACCTCAGAATCCATCGGACCCTGTGCTG**T**-3'	5'-**CTAGA**CAGCACAGGGTCCGATGGATTCTGAGGTGGTTTTGCATA**GAGCT**-3'
**Ctsl Target Region**	5'-ATCGCCGTGTAATTCTAGTTGTTTGGGTATGGAAGGCTACATCAAA-3'	5'-AGACTCGAGGCTAGCGAGCTCGTTTATGGGACACTGAGGTGAAATC-3'
**Ctsl Target Site**	5'-**C**TCAGCTTAAAACTGACCAAACCCTTATTGAG**T**-3'	5'-**CTAGA**CTCAATAAGGGTTTGGTCAGTTTTAAGCTGA**CAGCT**-3'
**Ctsl Mutant Target Site**	5'-**C**TCAGCTTAAAACTGACCAAACCCTTATGAGG**T**-3'	5'-**CTAGA**CCTCATAAGGGTTTGGTCAGTTTTAAGCTGA**GAGCT**-3'
**Ewsr1 Target Region**	5'-ATCGCCGTGTAATTCTAGTTGTTTCACTGCTACAGTCACCACAA-3'	5'-AGACTCGAGGCTAGCGAGCTCGTTTCCAGACTCCTGCCCATAAAC-3'
**Ewsr1 Target Site**	5'-**C**CAGTCACCGCACCTCCATCTTATCCTC**T**-3'	5'-**CTAGA**GAGGATAAGATGGAGGTGCGGTGACTG**GAGCT**-3'
**Ewsr1 Mutant Target Site**	5'-**C**CAGTCACCGCAAAGCCATCTTATCCTC**T**-3'	5'-**CTAGA**GAGGATAAGATGGCTTTGCGGTGACTG**GAGCT**-3'
**Fus Target Region**	5'-ATCGCCGTGTAATTCTAGTTGTTTCGGTCGTCTGGAACTTTGT-3'	5'-AGACTCGAGGCTAGCGAGCTCGTTTGTTGACCATAACCTCCACTCTG-3'
**Fus Target Site**	5'-**C**ACGACTATACCCAACAAGCAACTCAAAGCTA**T**-3'	5'-**CTAGA**TAGCTTTGAGTTGCTTGTTGGGTATAGTCGT**GAGCT**-3'
**Fus Mutant Target Site**	5'-**C**ACGACTATACCCAACAAGCGGACCAAAGCTA**T**-3'	5'-**CTAGA**TAGCTTTGGTCCGCTTGTTGGGTATAGTCGT**GAGCT**-3'
**Ifitm3 Target Region**	5'-ATCGCCGTGTAATTCTAGTTGTTTAGAGCAGTTTCATCAAGCCA-3'	5'-AGACTCGAGGCTAGCGAGCTCGTTTTCGGAATCCTCTATTAAGTGTGAAG-3'
**Ifitm3 Target Site**	5'-**C**CTCCGCACCATGAACCACACTTCTCAA**T**-3'	5'-**CTAGA**TTGAGAAGTGTGGTTCATGGTGCGGAG**GAGCT**-3'
**Ifitm3 Mutant Target Site**	5'-**C**CTCCGCACCATGAAGTGAAGGACT**T**-3'	5'-**CTAGA**AGTCCTTCACTTCATGGTGCGGAG**GAGCT**-3'
**Phc3 Target Region**	5'-ATCGCCGTGTAATTCTAGTTGTTTCTCACAGAATGGCTCACCAA-3'	5'-AGACTCGAGGCTAGCGAGCTCGTTTGGAGGAGACACCACTACAGA-3'
**Phc3 Target Site**	5'-**C**ATTCCTCTTCATTCACCACCTCCTAAAGTTTCCCATCATCAGCTGCTATTA**T**-3'	5'-**CTAGA**TAATAGCAGCTGATGATGGGAAACTTTAGGAGGTGGTGAATGAAGAGGAAT**GAGCT**-3'
**Phc3 Mutant Target Site**	5'-**C**ATTCCTCTTCATTCACGGGATCCTAAAGTTTCCCATCATCAGCTGCTATT**T**-3'	5'-**CTAGA**AATAGCAGCTGATGATGGGAAACTTTAGGATCCCGTGAATGAAGAGGAAT**GAGCT**-3'
**Foxj3 Target Region**	5'-ATCGCCGTGTAATTCTAGTTGTTTACAGCATGGCTTGATGAATTTAC-3'	5'-AGACTCGAGGCTAGCGAGCTCGTTTAAGTATGTACACATCACCCTTTCA-3'
**Foxj3 Target Site**	5'-**C**TAGAGACTTGGGGTAAGGACTAACAAACATACATACACAGATG**T**-3'	5'-**CTAGA**CATCTGTGTATGTATGTTTGTTAGTCCTTACCCCAAGTCTCTA**GAGCT**-3'
**Kdm5b Target Region**	5'-ATCGCCGTGTAATTCTAGTTGTTTAGGAGGACTCTGAGGATGAA-3'	5'-AGACTCGAGGCTAGCGAGCTCGTTTGCAAACTCCAATACCTTCGTAATC-3'
**Kdm5b Target Site**	5'-**C**CTGTACACGGTGCTATTTCTATTCCTTATGGGA**T**-3'	5'-**CTAGA**TCCCATAAGGAATAGAAATAGCACCGTGTACAG**GAGCT**-3'
**Trp53inp1 Target Region**	5'-ATCGCCGTGTAATTCTAGTTGTTTCGCAGCTACCTCAGCAC-3'	5'-AGACTCGAGGCTAGCGAGCTCGTTTACTGAAGGAAGCAGGAATCAC-3'
**Trp53inp1 Target Site**	5'-**C**AAACACTGTGCACTTTAATCTCTCGTGGAATTCTCGGGTGCCAAGGAA CTCCAGTCACCGATGTATCTCGTATGCCGTCT**T**-3'	5'-**CTAGA**AGACGGCATACGAGATACATCGGTGACTGGAGTTCCTTGGCACCC GAGAATTCCACGAGAGATTAAAGTGCACAGTGTTT**GAGCT**-3'
