# Supplementary material for: Identification of murine gammaherpesvirus 68 miRNA-mRNA hybrids reveals miRNA target conservation among gammaherpesviruses including host translation and protein modification machinery
Source: PLoS Pathog. 2019 Aug 8;15(8):e1007843. doi: 10.1371/journal.ppat.1007843 (PMC6687095; doi:10.1371/journal.ppat.1007843)
Supplement: S2 Table — Primers used for PCR amplification of target transcripts are indicated. (PDF) [file ppat.1007843.s007.pdf]

Table S2

Sequences of primers used for qRT-PCR analyses.

Gene	Sense Primer	Antisense Primer
Arid1a	5'-TGAACCTACAACGCCCTGAC-3'	5'-GCCGCTGCAGCTAAAGGTTA-3'
Ctsl	5'-GTGGACTGTTCTCACGCTCA-3'	5'-TCCGAGTCCAGACCTCCATT-3'
Ewsr1	5'-AGGGGAAGAGGGGGATTTGA-3'	5'-GAAGCCACCTCGCTCTCCAG-3'
Fus	5'-GTAAGGGCTTCAGTTTGTGGC-3'	5'-TGGCTCCCAAGTTCTCACAA-3'
Ifitm3	5'-CTGAACATCAGCACCTTGGTC-3'	5'-AGTGTGAAGGTTTTGAGCGTT-3'
Phc3	5'-GATGTCGGCATCTCCTCCAG-3'	5'-ACCTGGCCCTGGGATAGAAT-3'
Foxj3	5'-CAGTGCCACAGGTAGTAATTC-3'	5'-GGTAAACCATGCGGTCTATG-3'
Kdm5b	5'-CCAGTGTGTGGAGCATTATC-3'	5'-CGCTACCACAACGTCTAATAC-3'
Trp53inp1	5'-GGTCTCAGTGAGGCGAGTTG-3'	5'-CATTTCTGTGCCCGTGAGTC-3'
Gapdh	5'-CATGGCCTTCCGTGTTCCTA-3'	5'-CCTGCTTCACCACCTTCTTGAT-3'
