# Supplementary material for: Identification of murine gammaherpesvirus 68 miRNA-mRNA hybrids reveals miRNA target conservation among gammaherpesviruses including host translation and protein modification machinery
Source: PLoS Pathog. 2019 Aug 8;15(8):e1007843. doi: 10.1371/journal.ppat.1007843 (PMC6687095; doi:10.1371/journal.ppat.1007843)
Supplement: S3 Table — Numbers indicate the total reads or hybrids recovered in each qCLASH replicate for each sample group (lytic, reactivation or latency). Total number of virus plus host miRNA-containing hybrids (“total miRNA hybrids”) is indicated and reflects a proportion of all total RNA-RNA hybrids (“total number of hybrids”) obtained in Hyb output. (PDF) [file ppat.1007843.s008.pdf]

Table S3

Number of reads and hybrids identified within biological replicates and individual infection groups.

Lytic				
	Replicate 1	Replicate 2	Replicate 3	Total
Total Number of Reads	33,155,072	18,657,088	24,443,598	76,255,758
Total Number of Hybrids	75,222	145,353	133,142	353,717
Total miRNA Hybrids	31,277	67,120	57,984	156,381
Total Host miRNA Hybrids	26,946	59,127	50,501	136,574
Total Viral miRNA Hybrids	4,331	7,993	7,483	19,807

Reactivation				
	Replicate 1	Replicate 2	Replicate 3	Total
Total Number of Reads	36,040,560	17,794,998	20,190,940	74,026,498
Total Number of Hybrids	129,172	83,977	113,026	326,175
Total miRNA Hybrids	43,425	25,936	39,812	109,173
Total Host miRNA Hybrids	36,476	21,801	33,980	92,257
Total Viral miRNA Hybrids	6,949	4,135	5,832	16,916

Latency				
	Replicate 1	Replicate 2	Replicate 3	Total
Total Number of Reads	9,038,009	6,467,044	8,322,503	23,827,556
Total Number of Hybrids	20,906	18,403	24,934	64,243
Total miRNA Hybrids	8,146	9,100	11,945	29,191
Total Host miRNA Hybrids	7,302	8,108	10,629	26,039
Total Viral miRNA Hybrids	844	992	1,316	3,152
