# Supplementary material for: Identification of murine gammaherpesvirus 68 miRNA-mRNA hybrids reveals miRNA target conservation among gammaherpesviruses including host translation and protein modification machinery
Source: PLoS Pathog. 2019 Aug 8;15(8):e1007843. doi: 10.1371/journal.ppat.1007843 (PMC6687095; doi:10.1371/journal.ppat.1007843)
Supplement: S4 Table — For each host mRNA selected for validation, columns indicate: the miRNA contained within the hybrid, the number of different binding sites for the miRNA located within the transcript, the total number of interactions observed for this mRNA-miRNA combination, the mRNA transcript region targeted by miRNA binding, the complementarity of binding within the miRNA seed sequence (nt 2–8), the extent of complementarity downstream of the seed sequence, and whether or not the target is found within previously published EBV and KSHV CLIP data sets. In addition, a visual representation of the miRNA-mRNA interaction is presented. (PDF) [file ppat.1007843.s009.pdf]

Table S4

Binding characteristics of MHV68 miRNA-host mRNA hybrids selected for validation.

<table>
<tr><th>Transcript</th><th>miRNA</th><th>Binding Sites</th><th>Number of Interactions</th><th>Region Targeted</th><th>Seed binding*</th><th>3' Binding+</th><th>Visual Representation of Binding^</th><th>EBV Target?</th><th>KSHV Target?</th></tr>
<tr><td>Arid1a</td><td>6-3p</td><td>1</td><td>130</td><td>3' UTR</td><td>seed 7m1</td><td>strong</td><td>UGAAACUGUG...UGAGGUGGUUU
|||||| : |||||||||||
CAUUUGACCUAAGACUCCACCAAA</td><td>✓</td><td>✓</td></tr>
<tr><td>Ctsl</td><td>8-5p</td><td>1</td><td>112</td><td>3' UTR</td><td>other</td><td>strong</td><td>CACUCACUGGGGGUUUGGUC
|||| |::|||||||||
GAGUUAUUCCCAAACCAG</td><td>✓</td><td></td></tr>
<tr><td>Ewsr1</td><td>7-5p</td><td>1</td><td>91</td><td>CDS</td><td>seed 7</td><td>moderate</td><td>AAAGGUGGAGGUGCGGUAACCU
|||:||||||||||||||
AUUCUACCUCCACGCCACU</td><td>✓</td><td>✓</td></tr>
<tr><td>Fus</td><td>5-5p</td><td>1</td><td>26</td><td>CDS</td><td>seed 6</td><td>moderate</td><td>AGAGUUGAGAUC.GGGUCGUCU
|||||| ||||
ACUCAACGAACAACCCAUAUCA</td><td>✓</td><td>✓</td></tr>
<tr><td>Ifitm3</td><td>7-5p</td><td>2</td><td>4</td><td>5' UTR-CDS</td><td>seed 7</td><td>moderate</td><td>AAAG.GUGGA....GGUGCGGUAACC
||| |||| |||||||
CUUCACACCAAGUACCACGCCUC</td><td>✓</td><td></td></tr>
<tr><td>Phc3</td><td>6-3p</td><td>1</td><td>5</td><td>CDS</td><td>seed 6</td><td>strong</td><td>UGAAACUGUGUGAGGUGGUUUU
|||||| |: ||||||||
CCUUUGAAAUCCUCCACCACUU</td><td>✓</td><td>✓</td></tr>
<tr><td>Foxj3</td><td>12-3p</td><td>2</td><td>5</td><td>3' UTR</td><td>other</td><td>strong</td><td>UUUGGUGUGGGAGUCCU.ACCCCUU
||| |||||| |||||
CAAACAA..UCAGGAAUGGGGUU</td><td>✓</td><td>✓</td></tr>
<tr><td>Kdm5b</td><td>1-3p</td><td>3</td><td>71</td><td>3' UTR</td><td>seed 7</td><td>weak</td><td>UAGAAAUGGC..CGUACUUCCUUU
|||||||:|| |||
ATCUUUAUCGUGGCACAU</td><td>✓</td><td>✓</td></tr>
<tr><td>Trp53inp1</td><td>5-5p</td><td>1</td><td>5</td><td>5' UTR</td><td>other</td><td>moderate</td><td>AGAGUUGAGAUC.GGGTCGUC
||||| :::|| |:
GCUCUCUAAUUUCA.CGUG</td><td></td><td></td></tr>
</table>

* Indicates the complementarity within the miRNA seed sequence (miRNA nt 2-8): "seed 6" indicates complementarity of 6 consecutive nt within the seed sequence, "seed 7" indicates complementarity of all 7 nt, "seed 7m1" indicates 1 mismatch within the 7 nt seed, "other" indicates complementarity to less than 6 nt.

+ Indicates the extent of complementarity within the 3' portion of the miRNA (those nts downstream of the seed sequence).

^ Top strand is miRNA sequence, bottom strand is mRNA target sequence. Vertical lines between nucleotides indicates base pairing and dashed lines represent G:U wobble pairing. Dots between nucleotides represent a stretch of nucleotides on the opposing side which contains a bulge.
