# Supplementary material for: Identification of murine gammaherpesvirus 68 miRNA-mRNA hybrids reveals miRNA target conservation among gammaherpesviruses including host translation and protein modification machinery
Source: PLoS Pathog. 2019 Aug 8;15(8):e1007843. doi: 10.1371/journal.ppat.1007843 (PMC6687095; doi:10.1371/journal.ppat.1007843)
Supplement: S5 Table — Targets of MHV68 miRNAs found to be enriched within the top 15 canonical host signaling pathways, as identified by Ingenuity Pathway Analysis. (PDF) [file ppat.1007843.s010.pdf]

Table S5

MHV68 miRNA targets enriched in canonical host signaling pathways.

Pathway	Targets of MHV68 miRNAs
ATM signaling	ABL1,ATF1,ATF4,ATM,ATR,BID,BLM,BRCA1,CBX1,CBX5,CCNB1,CDC25A,CDC25C,CDK1,CDK2,CDKN1A,CHEK1,CREB1,CREB3,CREBBP,H2AFX,HERC2,HIST1H4J,HP1BP3,JUN,KAT5,MAPK9,MAPK10,MAPK11,MAPK14,MDM2,MDM4,NBN,NFKBIA,PPM1L,PPP2CA,PPP2CB,PPP2R1A,PPP2R1B,PPP2R2A,PPP2R2C,PPP2R5A,PPP2R5D,PPP2R5E,PTPA,RAD17,RAD50,RNF8,RNF168,SMC2,SMC3,SMC1A,TDP1,TLK1,TLK2,TOPBP1,TP53,TP53BP1,TRIM28,TRRAP,USP7
B cell receptor signaling	ABL1,AKT1,AKT2,AKT3,APBB1IP,ATF4,ATM,BCL6,BCL2L1,BLNK,BTK,Calm1,CAMK4,CD22,CD79A,CD79B,CHUK,CREB1,CREB3,CREBBP,CSK,EBF1,EGR1,ELK1,ETS1,FCGR2A,FLT3,FRS2,GRB2,GSK3A,GSK3B,INPP5D,INPP5F,INPPL1,IRS2,JUN,KRAS,LYN,MAP2K1,MAP2K2,MAP2K3,MAP2K7,MAP3K1,MAP3K2,MAP3K4,MAP3K5,Map3k7,MAP3K10,MAP3K11,MAPK1,MAPK3,MAPK9,MAPK11,MAPK14,MEF2C,NFAT5,NFATC1,NFATC3,NFKB1,NFKB2,NFKBIA,NFKBIB,NFKBID,NRAS,PAG1,PAX5,PDPK1,PIK3C3,PIK3C2A,PIK3C2B,PIK3CB,PIK3CG,PIK3R1,PLCG2,POU2F2,PPP3CA,PPP3CB,PPP3R1,PRKCB,PTEN,PTK2,PTK2B,PTPN6,PTPN11,PTPRC,RAC1,RAC2,RAP1A,RAP1B,RELA,RPS6KB1,RRAS,SHC1,SOS1,SOS2,SYNJ1,TCF3,VAV1,VAV2
chronic myeloid leukemia	ABL1,AKT1,AKT2,AKT3,ATM,BCL2L1,CDK4,CDK6,CDKN1A,CDKN1B,CDKN2A,CHUK,CRK,CRKL,CTBP1,E2F1,E2F2,E2F3,E2F4,E2F6,E2F7,E2F8,FLT3,FRS2,GRB2,HDAC1,HDAC2,HDAC5,HDAC7,HDAC9,HDAC10,IRS2,KRAS,MAP2K1,MAP2K2,MAPK1,MAPK3,MDM2,MECOM,MYC,NFKB1,NFKB2,NRAS,PA2G4,PIK3C3,PIK3C2A,PIK3C2B,PIK3CB,PIK3CG,PIK3R1,PTPN11,RAP1A,RAP1B,RB1,RBL1,RELA,RRAS,SIN3A,SMAD4,SOS1,SOS2,STAT5A,STAT5B,TFDP1,TGFB1,TGFBR2,TP53
cyclins and cell cycle regulation	ABL1,ATM,ATR,BTRC,CCNA2,CCNB1,CCND3,CCNE2,CCNH,CDC25A,CDK1,CDK2,CDK4,CDK6,CDK7,CDKN1A,CDKN1B,CDKN2A,CDKN2C,CUL1,E2F1,E2F2,E2F3,E2F4,E2F6,E2F7,E2F8,FBXL5,GSK3B,HDAC1,HDAC2,HDAC5,HDAC7,HDAC9,HDAC10,MYT1,PA2G4,PPM1L,PPP2CA,PPP2CB,PPP2R1A,PPP2R1B,PPP2R2A,PPP2R2C,PPP2R5A,PPP2R5D,PPP2R5E,PTPA,RB1,SIN3A,SKP2,TFDP1,TGFB1,TP53,WEE1
DNA methylation and transcription	ARID4B,CHD3,CHD4,DNMT1,DNMT3A,H3F3A/H3F3B,HDAC1,HDAC2,HIST1H4A,HIST1H4B,HIST1H4C,HIST1H4H,HIST1H4I,HIST1H4J,HIST1H4K,HIST1H4L,HIST2H4A,HIST2H4B,HIST4H4,MBD3,MECP2,MTA1,MTA2,RBBP4,RBBP7,SAP18,SAP30,SAP130,SIN3A,SUDS3
EIF2 signaling	ACTB,AGO1,AGO2,AKT1,AKT2,AKT3,ATF4,ATF5,ATM,BCL2,CDK11A,DDIT3,EIF5,EIF1AX,EIF1AY,EIF2AK2,EIF2B2,EIF2B4,EIF2B5,EIF2S1,EIF2S2,EIF2S3,EIF3A,EIF3B,EIF3C,EIF3D,EIF3E,EIF3F,EIF3G,EIF3H,EIF3I,EIF3J,EIF3K,EIF3L,EIF3M,EIF4A1,EIF4A2,EIF4A3,EIF4E,EIF4G1,EIF4G2,EIF4G3,EIF5B,FAU,FLT3,FRS2,GRB2,GSK3B,HSPA5,IRS2,KRAS,MAP2K1,MAP2K2,MAPK1,MAPK3,MYC,NOX4,NRAS,PABPC1,PAIP1,PDPK1,PIK3C3,PIK3C2A,PIK3C2B,PIK3CB,PIK3CG,PIK3R1,PPP1CA,PPP1CB,Ppp1cc,PPP1R15A,PTBP1,PTPN11,RAP1A,RAP1B,RPL3,RPL4,RPL5,RPL6,RPL7,RPL8,RPL9,RPL11,RPL12,RPL13,RPL14,RPL15,RPL17,RPL18,RPL19,RPL21,RPL22,RPL23,RPL24,RPL26,RPL27,RPL28,RPL30,RPL31,RPL35
EIF4 and p70S6K signaling	AGO1,AGO2,AKT1,AKT2,AKT3,ATM,EIF1AX,EIF1AY,EIF2B2,EIF2B4,EIF2B5,EIF2S1,EIF2S2,EIF2S3,EIF3A,EIF3B,EIF3C,EIF3D,EIF3E,EIF3F,EIF3G,EIF3H,EIF3I,EIF3J,EIF3K,EIF3L,EIF3M,EIF4A1,EIF4A2,EIF4A3,EIF4E,EIF4EBP1,EIF4EBP2,EIF4G1,EIF4G2,EIF4G3,FAU,FLT3,FRS2,GRB2,IRS2,ITGA4,ITGA5,ITGB1,KRAS,MAP2K1,MAP2K2,MAPK1,MAPK3,MAPK11,MAPK14,MKNK1,NRAS,PABPC1,PAIP1,PAIP2,PDPK1,PIK3C3,PIK3C2A,PIK3C2B,PIK3CB,PIK3CG,PIK3R1,PPM1L,PPP2CA,PPP2CB,PPP2R1A,PPP2R1B,PPP2R2A,PPP2R2C,PPP2R5A,PPP2R5D,PPP2R5E,PTPA,PTPN11,RAP1A,RAP1B,RPS2,RPS3,RPS5,RPS6,RPS7,RPS8,RPS9,RPS10,RPS11,RPS13,RPS14,RPS15,RPS16,RPS17,RPS19,RPS20,RPS21,RPS23,RPS24,RPS25,RPS26,RPS28,RPS29
ERK MAPK signaling	ARAF,ATF1,ATF4,ATM,BRAF,CREB1,CREB3,CREBBP,CRK,CRKL,DUSP1,DUSP2,DUSP4,DUSP6,EIF4E,EIF4EBP1,ELF1,ELF2,ELF4,ELK1,ELK3,ETS1,FLT3,FRS2,FYN,GRB2,H3F3A/H3F3B,HIST1H3C,HSPB7,IRS2,ITGA4,ITGA5,ITGB1,KRAS,LAMTOR3,MAP2K1,MAP2K2,MAPK1,MAPK3,MAPKAPK5,MKNK1,MKNK2,MYC,NFATC1,NRAS,PAK2,PAK3,PIK3C3,PIK3C2A,PIK3C2B,PIK3CB,PIK3CG,PIK3R1,PLA2G6,PLA2G12A,PLCG1,PLCG2,PPARG,PPM1L,PPP1CA,PPP1CB,PPP1R7,PPP1R10,PPP1R12A,PPP1R14B,PPP2CA,PPP2CB,PPP2R1A,PPP2R1B,PPP2R2A,PPP2R2C,PPP2R5A,PPP2R5D,PPP2R5E,PRKACB,PRKAG2,PRKAR1A,PRKAR2A,PRKCB,PRKCD,PRKCI,PTK2,PTK2B,PTPA,PTPN11,PXN,RAC1,RAC2,RAP1A,RAP1B,RAPGEF1,RAPGEF3,RRAS,SHC1,SOS1,SOS2,SRF,STAT1,STAT3,TLN1
estrogen receptor signaling	CCNC,CCNH,CDK7,CDK8,CREBBP,CTBP1,DDX5,EP300,ERCC2,ERCC3,GRB2,GTF2A1,GTF2E1,GTF2F1,GTF2F2,GTF2H1,GTF2H2,GTF2H5,H3F3A/H3F3B,HIST1H3C,HNRNPD,KRAS,MAP2K1,MAP2K2,MAPK1,MAPK3,MED1,MED12,MED13,MED14,MED15,MED16,MED23,MED24,MED30,MED12L,MED13L,NCOA1,NCOA2,NCOA3,NCOR1,NCOR2,NR3C1,NRAS,NRIP1,PCK2,PELP1,PGR,PHB2,POLR2A,POLR2B,POLR2C,POLR2E,POLR2J,POLR2L,PRKDC,RAP1A,RAP1B,RRAS,SHC1,SMARCA4,SOS1,SOS2,SPEN,SRA1,TAF1,TAF5,TAF6,TAF7,TAF10,TAF12,TAF15,TAF4B,TAF5L,TAF6L,TBP,THRAP3,TRRAP
molecular mechanisms of cancer	ABL1,AKT1,AKT2,AKT3,APC,APH1A,ARHGEF1,ARHGEF2,ARHGEF6,ARHGEF7,ARHGEF12,ARHGEF17,ARHGEF18,ATM,ATR,AURKA,AXIN1,BAK1,BAX,BBC3,BCL2,BCL2L1,BCL2L11,BID,BIRC2,BIRC3,BMPR2,BMPR1A,BRAF,BRCA1,CASP3,CASP7,CBL,CCND3,CCNE2,CDC25A,CDC25B,CDC25C,CDK1,CDK2,CDK4,CDK6,CDK7,CDK8,CDK12,CDK13,CDK15,CDK16,CDK17,CDK19,CDK11A,CDKN1A,CDKN1B,CDKN2A,CDKN2C,CHEK1,CREBBP,CRK,CTNNB1,CYCS,DIABLO,E2F1,E2F2,E2F3,E2F4,E2F6,E2F7,E2F8,ELK1,EP300,FAS,FLT3,FNBP1,FRS2,FYN,GNA13,GNAI2,GNAI3,GNAQ,GNAS,GNAZ,GRB2,GSK3A,GSK3B,HIF1A,HIPK2,IRS2,ITGA4,ITGA5,ITGB1,JAK1,JUN,KRAS,LAMTOR3,LEF1,LRP6,MAP2K1,MAP2K2,MAP2K3,MAP3K5
mTOR signaling	AKT1,AKT2,AKT3,AKT1S1,ATM,DDIT4,DGKZ,EIF3A,EIF3B,EIF3C,EIF3D,EIF3E,EIF3F,EIF3G,EIF3H,EIF3I,EIF3J,EIF3K,EIF3L,EIF3M,EIF4A1,EIF4A2,EIF4A3,EIF4B,EIF4E,EIF4EBP1,EIF4G1,EIF4G2,EIF4G3,FAU,FKBP1A,FLT3,FNBP1,FRS2,GRB2,HIF1A,HMOX1,IRS2,KRAS,MAPK1,MAPK3,NAPEPLD,NRAS,PDPK1,PIK3C3,PIK3C2A,PIK3C2B,PIK3CB,PIK3CG,PIK3R1,PLD3,PPM1L,PPP2CA,PPP2CB,PPP2R1A,PPP2R1B,PPP2R2A,PPP2R2C,PPP2R5A,PPP2R5D,PPP2R5E,PRKAA1,PRKAB1,PRKAG2,PRKCB,PRKCD,PRKCI,PRR5,PTPA,PTPN11,RAC1,RAP1A,RAP1B,RHOA,RHOB,RHOG,RHOH,RHOQ,RHOT2,RICTOR,RPS2,RPS3,RPS5,RPS6,RPS7,RPS8,RPS9,RPS10,RPS11,RPS13,RPS14,RPS15,RPS16,RPS17,RPS19,RPS20,RPS21,RPS23,RPS24,RPS25
PI3K AKT signaling	AKT1,AKT2,AKT3,BCL2,BCL2L1,CDC37,CDKN1A,CDKN1B,CHUK,CTNNB1,EIF4E,EIF4EBP1,FOXO3,GRB2,GSK3A,GSK3B,GYS1,HSP90AA1,HSP90AB1,HSP90B1,INPP5D,INPP5F,INPPL1,ITGA4,ITGA5,ITGB1,JAK1,KRAS,LIMS1,MAP2K1,MAP2K2,MAP3K5,MAPK1,MAPK3,MCL1,MDM2,NFKB1,NFKB2,NFKBIA,NFKBIB,NFKBID,NRAS,PDPK1,PIK3CB,PIK3CG,PIK3R1,PPM1L,PPP2CA,PPP2CB,PPP2R1A,PPP2R1B,PPP2R2A,PPP2R2C,PPP2R5A,PPP2R5D,PPP2R5E,PTEN,PTPA,RAP1A,RAP1B,RELA,RPS6KB1,RRAS,SHC1,SOS1,SOS2,SYNJ1,TP53,TSC1,TSC2,YWHAB,YWHAE,YWHAG,YWHAH,YWHAQ,YWHAZ
protein ubiquitination	AMFR,ANAPC1,ANAPC4,ANAPC5,B2M,BAP1,BIRC2,BIRC3,BIRC6,BRCA1,BTRC,CBL,CDC20,CDC34,CUL1,CUL2,DNAJA1,DNAJB1,DNAJB2,DNAJB5,DNAJB6,DNAJB9,DNAJB11,DNAJB12,DNAJC2,DNAJC5,DNAJC7,DNAJC8,DNAJC9,DNAJC10,DNAJC11,DNAJC13,DNAJC16,DNAJC21,DNAJC25,DNAJC30,FBXW7,FZR1,HLAA,HSP90AA1,HSP90AB1,HSP90B1,HSPA2,HSPA4,HSPA5,HSPA8,HSPA9,HSPA14,HSPA12B,HSPA4L,HSPB7,HSPD1,HSPE1,MDM2,NEDD4L,PSMA1,PSMA3,PSMA4,PSMA7,PSMB1,PSMB5,PSMB6,PSMB8,PSMB10,PSMC1,PSMC2,PSMC3,PSMC5,PSMC6,PSMD1,PSMD2,PSMD4,PSMD5,PSMD6,PSMD7,PSMD8,PSMD11,PSMD12,PSMD13,PSMD14,PSME1,RPS27A,Sacs,SKP2,SMURF2,TAP1,TAP2,THOP1,TRAF6,TRAP1,UBA1,Ubb,UBC,UBE2B,UBE2D3,UBE2E1,UBE2F,UBE2G1,UBE2G2,UBE2H
sumoylation pathway	ARHGDIA,ARHGDIB,CBX4,CEBPA,CREBBP,CTBP1,DNMT3A,EP300,ETS1,FAS,FNBP1,GDI1,GDI2,HDAC1,HDAC2,JUN,KDM1A,MAP3K5,MAPK9,MAPK10,MDM2,MYB,NFKB1,NFKB2,NFKBIA,NR3C1,PCNA,PIAS1,PIAS4,RAN,RANGAP1,RCC1,RCOR1,RFC1,RFC2,RFC5,RHOA,RHOB,RHOG,RHOH,RHOQ,RHOT2,RNF4,SENP1,SENP2,SENP3,SENP5,SENP6,SENP7,SERBP1,SIRT1,SLC19A1,SMAD4,SP1,SP3,SUMO1,SUMO2,SUMO3,TDG,TP53,UBA2,UBE2I,XIAP,ZNF217
unfolded protein response	AMFR,ATF4,ATF6,BCL2,CALR,CANX,CD82,CEBPA,CEBPB,CEBPZ,DDIT3,DNAJA2,DNAJB9,EDEM1,ERN1,ERO1B,HSP90B1,HSPA2,HSPA4,HSPA5,HSPA8,HSPA9,HSPA14,INSIG1,MAP2K7,MAP3K5,MBTPS1,NFE2L2,OS9,P4HB,PDIA6,PPARG,PPP1R15A,SCAP,SEL1L,SREBF1,SREBF2,SYVN1,TRAF2,UBXN4,VCP,XBP1
